# Arabidopsis Plants Sense Non-self Peptides to Promote Resistance Against *Plectosphaerella cucumerina*

**DOI:** 10.3389/fpls.2020.00529

**Published:** 2020-05-08

**Authors:** Julia Pastor-Fernández, Jordi Gamir, Victoria Pastor, Paloma Sanchez-Bel, Neus Sanmartín, Miguel Cerezo, Víctor Flors

**Affiliations:** Metabolic Integration and Cell Signaling Laboratory, Plant Physiology Section, Unidad Asociada al Consejo Superior de Investigaciones Científicas (EEZ-CSIC)-Department of Ciencias Agrarias y del Medio Natural, Universitat Jaume I, Castellón, Spain

**Keywords:** systemin, induced resistance, Arabidopsis, LC-MS, *Plectoshaerella cucumerina*

## Abstract

Peptides are important regulators that participate in the modulation of almost every physiological event in plants, including defense. Recently, many of these peptides have been described as defense elicitors, termed phytocytokines, that are released upon pest or pathogen attack, triggering an amplification of plant defenses. However, little is known about peptides sensing and inducing resistance activities in heterologous plants. In the present study, exogenous peptides from solanaceous species, Systemins and HypSys, are sensed and induce resistance to the necrotrophic fungus *Plectosphaerella cucumerina* in the taxonomically distant species *Arabidopsis thaliana.* Surprisingly, other peptides from closer taxonomic clades have very little or no effect on plant protection. *In vitro* bioassays showed that the studied peptides do not have direct antifungal activities, suggesting that they protect the plant through the promotion of the plant immune system. Interestingly, tomato Systemin was able to induce resistance at very low concentrations (0.1 and 1 nM) and displays a maximum threshold being ineffective above at higher concentrations. Here, we show evidence of the possible involvement of the JA-signaling pathway in the Systemin-Induced Resistance (Sys-IR) in Arabidopsis. Additionally, Systemin treated plants display enhanced *BAK1* and *BIK1* gene expression following infection as well as increased production of ROS after PAMP treatment suggesting that Systemin sensitizes Arabidopsis perception to pathogens and PAMPs.

## Introduction

Plants are constantly challenged by changes in their environment, such as biotic and abiotic stresses. To respond to biotic challenges, such as chewing insects or pathogen attack, plants have developed complex strategies that allow them to mount a proper defense response. Plants can sense pathogens by recognizing the so-called pathogen-associated molecular patterns (PAMPs), which are exogenous molecules that belong to specific classes of microbes, such as flagellin (Flg22) and Elf18 from bacteria or chitin from fungi. PAMPs are recognized by membrane pattern recognition receptors (PRRs), triggering a first layer of inducible plant defense referred to as PAMP-triggered immunity (PTI) that includes reactive oxygen species (ROS) and Ca^2+^ burst, mitogen-activated protein kinases (MAPKs) activation, phytohormones production and transcriptomic and metabolomic reprogramming (Saijo et al., 2018; Hou et al., 2019).

Plants are also able to recognize host-derived molecules that are released from disrupted cells after pest or pathogen attack and bind to PRRs on intact cells, triggering the amplification of immune signaling. These molecules are known as damage-associated molecular patterns (DAMPs) and include, on the one hand, cell wall fragments that are released after cellular damage caused, for example, by herbivores and, on the other hand, peptide molecules that are released and rapidly activated upon pest or pathogen challenge and cause the amplification of immune signaling (Hou et al., 2019).

Although many peptides have been described as DAMPs, recent studies include these peptides in a new classification. Classic DAMPs are cell debris that are passively released after a cellular disruption and are usually components of the cell wall, such as oligogalacturonides (OGs) and xyloglucan oligosaccharides. Nevertheless, peptides are usually actively synthesized, processed and released by cells under a stress situation that does not include cell damage; these peptides are secondary endogenous danger signals, also named phytocytokines due to their similarity to mammalian cytokines (Gust et al., 2017).

Exposure to danger signals, such as PAMPs, DAMPs or phytocytokines, as well as many other stimuli, produces an alarm state in the plant, enhancing defense capacity locally and systemically that protects the plant against future attack (Gust et al., 2017; Yu et al., 2017; Hou et al., 2019). This state is called induced resistance (IR) and can be triggered by pathogenic and non-pathogenic microbes, herbivores and chemicals, leading to systemic acquired resistance (SAR), or by plant beneficial microbes, including plant growth-promoting rhizobacteria and fungi, leading induced systemic resistance (ISR) (Pieterse et al., 2014). The state of induced resistance is characterized by the rapid activation of latent defense mechanisms, for instance, the production of antimicrobial proteins, and confers protection against a broad spectrum of threats (Pieterse et al., 2014).

An increasing number of plant peptides have been described as defense elicitors. These peptides are released upon pest or pathogen attack and usually derived from the processing of larger precursor proteins, secreted into the extracellular space and bind to specific membrane receptors, triggering a cascade of plant defenses and causing an amplification of the plant immune response (Yamaguchi and Huffaker, 2011; Albert, 2013).

Systemin was the first signaling peptide described in plants (Pearce et al., 1991). Systemin is an 18 aa peptide found in tomato plants that is part of in a 200 aa precursor protein, Prosystemin. Systemin is released upon wounding or herbivory and induces the accumulation of protease inhibitors (PIs) in local and systemic leaves and volatile signaling that attract natural predators of the pest (Corrado et al., 2007). There is also evidence of the role of Systemin in defense against pathogenic fungi (De la Noval et al., 2007; Coppola et al., 2015, 2019). The hydroxyproline-rich systemins (HypSys) are peptides found in tomato and tobacco that trigger physiological responses that are similar to those triggered by tomato Systemin (Pearce et al., 2001; Pearce and Ryan, 2003). In Arabidopsis, elicitor peptides (Peps) were described as endogenous amplifiers of innate immunity that induce the transcription of defense-related genes, such as defensin *PDF1.2* and *PR1*, and activate the synthesis of reactive oxygen species (ROS; e.g., H_2_O_2_) (Huffaker et al., 2006; Klauser et al., 2013). AtPep1 participates in plant resistance against several pathogens, including *Botrytis cinerea, Pseudomonas syringae* pv. DC3000 and *Phytophthora infestans* (Huffaker et al., 2006; Yamaguchi et al., 2010; Liu et al., 2013), and contributes to JA-mediated defense against herbivory (Klauser et al., 2015). Another family of peptides, PAMP-induced peptides (PIPs), were identified in Arabidopsis and are induced by pathogens and elicitors. More specifically, when PIP1 and PIP2 are externally applied, they lead to enhanced immune responses and resistance to *Pseudomonas syringae* and *Fusarium oxysporum* (Hou et al., 2014). Likewise, three short peptides from Soybean, GmPep914, GmPep890, and GmSubPep, were found to alkalinize the cellular media and induce pathogen-related genes, such as *Chitinase 1* and *Chalcone Synthas*e, and genes involved in phytoalexin synthesis and production (Pearce et al., 2010; Yamaguchi et al., 2011).

Some peptides that were initially thought to be involved in different physiological events have been later found to have a role in defense responses. The Arabidopsis GRIM RIPER peptide (GRIp) is involved not only in the response to ozone but also in the resistance to bacterial pathogen PstDC3000 (Wrzaczek et al., 2009). Likewise, the IDA-LIKE 6 (IDL6) mature peptide was studied for its role in controlling floral organ abscission and lateral root emergence and was later found to be involved in the mediation of Arabidopsis susceptibility to *Pst* DC3000 (Wang et al., 2017). The peptides from rapid alkalinization factors (RALFs) were shown to positively and negatively regulate plant immunity through the RLK Feronia (FER) receptor (Stegmann et al., 2017). Recently, the plant pentapeptide, phytosulfokine (PSK), was found to enhance auxin-dependent immune responses through cytosolic Ca^2+^ signaling in tomato (Zhang et al., 2018).

Interestingly, some studies have reported peptide sensing and signaling in heterologous plant species. Although a report claims that tobacco cells do not respond to exogenous systemin treatment (Scheer et al., 2003), a later study showed that tobacco calli and suspension cells responded to Systemin by both MAPK activation and weak-medium alkalinization (Malinowski et al., 2009). In addition, it was also reported that constitutive expression of the tomato prosystemin gene in tobacco considerably affected the synthesis of host proteins, several of which are involved in protection against pathogens (Rocco et al., 2008). On the other hand, tobacco cells transformed with the AtPep1 receptor PEPR1 responded to nanomolar concentrations of AtPep1, producing a strong alkalinization of the cell culture medium, suggesting a capacity of tobacco to activate Pep1 signaling (Yamaguchi et al., 2006). More surprisingly, Zhang et al. (2017), reported that tomato Systemin was sensed by Arabidopsis plants, leading to an inhibition of seedling root growth and the expression of the plant defensin *PDF1.2*. Moreover, the expression of the tomato prosystemin gene in Arabidopsis conferred resistance to the necrotrophic fungus *Botrytis cinerea* (Zhang et al., 2017).

These findings suggest that some plants may be able to sense exogenous peptides and that there could be a common receptor-mediated intracellular signaling pathway in response to peptides.

Small peptides have recently received attention since they are involved in almost all physiological plants processes. The vast agronomical potential of peptides is limited by the studies focused on plant species-self peptides. We tested whether exogenous treatment with peptides produced from different plant species are sensed and able to protect Arabidopsis plants. Hence, the goal of this study was to identify peptides from phylogenetically distant species with plant-resistance inducing activities against necrotrophic fungal pathogens.

## Materials and Methods

### Plant Material and Growth Conditions

Seeds of wild type *Arabidopsis thaliana* Col-0 ecotype were sterilized for 30 s with 70% ethanol, followed by 15 min of a 10% bleach solution, and finally, 4–5 washes with sterile distilled water to remove the sterilization solution. Sterile seeds were sown *in vitro* 24-well plates in medium containing 4.9 g/L basal Murashige and Skoog (1962) salt mixture, 1% sucrose and 6 g/L Agar and 5.7 of pH. The plates were placed in a growth chamber with 9 h light period at 24°C and 15 h of darkness at 18°C; a dark surface was placed beneath the plates.

For the mutant screenings, the same procedure was carried out. The mutant *sid2.1* (Nawrath and Métraux, 1999) was kindly provided by M. Nishimura (Stanford University, CA, United States), j*ar1* (Matthes et al., 2010) by Jurriaan Ton (University of Sheffield, United Kingdom), and *jin1* (Lorenzo et al., 2004) and *pad4.1* (Nishimura et al., 2003) were provided by Brigitte Mauch-Mani (University of Neuchâtel, Switzerland) and the mutant perp1 was obtained from SALK collection (SALK_059281) and previously described by Flury et al. (2013).

Tomato seeds (*Solanum lycopesicum* L. cv. Money Maker) were sterilized by 15 min shaking in a solution of 75% bleach containing 0.1% of Tween, followed by 4–5 washes with sterile distilled water to remove the sterilization solution. The seeds were sown in 100 ml pots containing 30 ml of solid MS medium (described above). The pots were then placed in a growth chamber with 16 h light period at 26°C and 8 h of darkness at 18°C; a dark surface was placed beneath the plates.

### Peptide Treatment, Pathogen Inoculation and Infection Quantification by Trypan Blue Staining

The plants were treated 2 weeks after sowing with a range of peptide concentrations from 0.1 to 20 nM (final concentration) by adding the peptides to the medium. Twenty four hours after peptide treatment, plants were challenged with 5 × 10^3^ spores/ml of *Plectosphaerella cucumerina* by drop inoculation (1 μl per leaf). In Arabidopsis plants, BABA was used as a positive control at a concentration of 1 ppm (1 mg/L) (Pastor et al., 2013).

For the infection quantification, the plants were collected 5 days after infection and dead cells were stained using trypan blue (Ton and Mauch-Mani, 2004). The infection levels were quantified by a disease rating, measured as a percentage of infected leaf surface according to a scale (0 = healthy leaves; 1 = leaves with less than 25% of diseased surface; 2 = leaves with 25–50%; 3 = leaves with 50–75% of diseased surface; 4 = leaves with more than 75% diseased surface). A minimum of 6 plants per condition and 4 leaves per plant were analyzed. All experiments were repeated a minimum of three times.

### Fungal Biomass Quantification

Infection quantification was also determined by measuring a fungal constitutive gene related to a plant constitutive gene. Arabidopsis tissue of plants treated either with water or 0.1 nM systemin was collected for DNA extraction 48 h after pathogen infection. For the DNA extraction, a simple and rapid protocol was followed (Edwards et al., 1991). A Quantitative Real-Time PCR (qPCR) was performed with a Maxima SYBR Green/ROX qPCR Master Mix (2X) (Thermo Scientific), using a StepOne instrument (Applied Biosystems). A ratio was calculated of the expression of *PcTUBULIN*, as a constitutive gene of *P. cucumerina*, relative to the expression of *AtUBIQUITIN21*, a constitutive gene of Arabidopsis, following the ΔCt method. Primer sequences are listed in Supplementary Table S1.

### *In vitro* Antifungal Assays

Sterile 12-well plates were filled with PDB1/2 medium containing the peptides at the concentration of 20 nM, the highest concentration used in the screenings. A solution with *Plectosphaerella cucumerina* spores was added to each well to a final concentration of 10^4^ spores/ml in each well, and the plates were placed in a shaker until the next day. To measure the fungal growth, absorbance at 600 nm was measured 24 h after pathogen inoculation. This method was adapted from Broekaert et al. (1990). A commercial fungicidal was used as a positive control of growth inhibition.

### ROS Production Measurement

H_2_O_2_ production after treatments was determined in leaf discs using a luminol-based assay as previously described (Torres et al., 2013). Two different experiments were performed. Firstly, to determine the ROS production in response to Systemin treatments, a group of leaf discs (6 mm diameter; *n* = 8) obtained from 6-week-old plants were stored with 150 ml of water. After 24 h the water was replaced by water (blanc) or Systemin at different concentrations (0.1, 1, 10, 100, and 1000 nM) in a 96-well titer plate (one disc/well) with a solution containing luminol (Sigma-Aldrich; 100 μM) and horseradish peroxidase (Sigma-Aldrich; 1 μg mL^–1^). Secondly, to test whether Systemin treated plants were sensitive to PAMPs, the leaf discs were maintained overnight either with water or with increasing concentrations of systemin (0.1, 1, 10, 100, and 1000 nM). Twenty four hours later, H_2_O_2_ production was triggered by adding 100 nM flg22 to the leaf discs. Plates were analyzed for 1 h using a Luminoskan 96 microplate luminometer (Thermo Fisher Scientific) and a signal integration time of 1.5 s. Luminescence was expressed in Relative Luminescence Units.

### Targeted HPLC-MS for Hormonal Analysis

For hormonal analyses, 120 mg of freeze-dried material sampled at 48 hpi was powdered in liquid nitrogen and homogenized with 1 ml of MeOH: H_2_O (0.01%HCOOH) (10:90). Crystal balls were added to each sample and tubes were placed in shaker during 2.5 min at 30 Hz. Then, samples were centrifuged and the supernatant was collected into a new tube.

A mix of internal standards with salicylic acid-d5 (SA-d5), dehydrojasmonic acid (dhJA), and jasmonate-isoleucine-d6 (JA-Ile-d6) was added to each sample. To quantify precisely, external calibration curves were prepared with each pure compound (quantification, SA-d5 for SA, dhJA for JA and JA-Ile-d6 for JA-Ile). The targeted hormonal analysis was performed in an Acquity ultraperformance liquid chromatography system (UPLC; Waters, Mildford, MA, United States) coupled to a triple quadrupole mass spectrometer (Xevo TQS, Waters Micromass, Manchester, United Kingdom). The column used for the LC separation was a UPLC Kinetex 2.6 μm EVO C18 100 Å, 2.1 × 50 mm (Phenomenex). Conditions and solvent gradients used in this chromatographic analysis were the same as described in Sánchez-Bel et al. (2018).

### RNA Extraction and RT-qPCR Analysis

Two days post-inoculation (48 hpi), the leaves were collected, powdered in liquid nitrogen and stored at −80°C. For the RNA extraction, 1 ml of Trizol was added to 100 mg of grounded leaves. After centrifugation, the supernatant was transferred to a new tube, and 0.22 ml of CHCl3 was added. The samples were centrifuged, and the supernatant was collected in a new tube; 0.35 ml of isopropanol, 0.35 ml of 0.8 M citrate and 1.2 mM NaCl were added and mixed vigorously. After centrifugation, the supernatant was removed, and the pellet was washed twice with 70% EtOH. The pellet was dried and dissolved in nuclease-free water.

The synthesis of cDNA was performed using a High Capacity cDNA Reverse Transcription Kit (Applied Biosystems). Quantitative Real-Time PCR (qPCR) was performed with a Maxima SYBR Green/ROX qPCR Master Mix (2X) (Thermo Fisher Scientific), using a StepOne instrument (Applied Biosystems).

The ΔCt method was used to analyze the gene expression data. The housekeeping genes *UBIQUITIN21 (At5g25760)* and *PP2A (At1g13320)* were used to normalize the expression values.

The sequences of the primers are shown in Supplementary Table S1.

### Peptide Extraction

One day after peptide treatment, the seedlings were collected, powdered with liquid nitrogen and stored at −80°C. Fresh material (250 mg) was homogenized in a tube with 1.5 ml of Phenol/TRIS and saturated (ACROS Organic, ref. 327125000) at pH 8. The suspension was incubated at room temperature for 20 min, crystal balls were added to each sample and the tubes were placed in a shaker for 2.5 min at 30 Hz.

The tubes were centrifuged 2 min at 21.900 RCF. After centrifugation, the liquid phase was filtered using a hydrophilic PVDF filter with a 25 mm diameter and a pore size of 0.45 μm (FILTER-LAB). After filtration, 6 volumes of pure cold acetone (Scharlau, AC0312, PharmPur^®^) were added to each sample, and the samples were stored overnight at −20°C.

The precipitate was recovered the next day and washed twice with cold acetone. The liquid phase was discarded, and the pellet was dried. The final residue was re-suspended in 500 μl of a solution of 0.1% HCOOH in H_2_O: acetonitrile (9:1, v/v) and injected into the TQS-MS/MS instrument (Xevo TQS, Waters Micromass, Manchester, United Kingdom).

### Reagents and Standards

Supergradient HPLC-grade acetonitrile was purchased from Scharlab (AC 0331). Formic acid was obtained from J.T. Baker (Deventer, Holland, 6037). Methanol (HPLC grade), and trypan blue were purchased from Sigma^1^. Peptide standards of Systemin, Pep1, HypSysI, HypSysII, HypSysII, PotSysI, PotSysII, PepSys, NishSys, Pep914, Pep890, and Systemin-P13AT17A were purchased from Biomatik^2^.

### Optimization of a Multi-Residue Targeted Quantitative LC-MS Method for Small Peptide Analysis

High-performance liquid chromatography (HPLC) was performed using a Waters Xevo TQ-S. A protocol that was adapted from Pastor et al. (2018) was followed. Aliquots of 20 μl were injected into the system through a reversed column Aeris PEPTIDE 3.6 μ XB-C18 (150 × 4.6 mm) from Phenomenex, at a flow rate of 0.3 ml min^–1^.

The peptides were eluted with a gradient of ACN (organic phase) and Milli-Q water containing 0.1% HCOOH (aqueous phase), starting with 5:95 (v/v), linearly increasing to 35:65 (v/v) over 10 min and plateauing at 95:5 (v/v) 1 min later. The gradient was maintained in isocratic conditions for 1 min before the column was left to equilibrate for 3 min in order to reach initial conditions, for a total of 15 min per sample. The effluents originating from the HPLC were introduced into a triple quadrupole mass spectrometer (Xevo TQS, Waters Micromass, Manchester, United Kingdom) equipped with T-Wave devices and an ESI interface operated in positive mode. The cone and desolvation gas was nitrogen. The nebulizer gas flow was set to 250 L h^–1^ and the desolvation gas flow at 1200 L h^–1^. For operation in tandem MS/MS mode, the collision gas was pure 99.995% argon (Praxair, Madrid, Spain), at a pressure of 4 × 10^–3^ bar in the collision cell. The desolvation gas temperature was 650°C, the source temperature was set to 150°C, and the capillary voltage was 3.2 kV. The mass spectrometer was set to multiple reaction monitoring (MRM) mode, and the data were acquired and processed using the MassLynx v4.1 software (Waters, Manchester, United Kingdom).

For the selection of the precursor and daughter ions of each peptide, peptide standards direct infusion was performed in a Waters Xevo TQ-S instrument, and masses showing the highest signal were selected for fragmentation and daughter ion characterization. Optimal conditions and appropriate cone and collision energies were determined to obtain the characteristic transitions for each peptide. Second, the retention time for each peptide was characterized by injecting aliquots of the standard peptides in a range of concentrations to construct calibration curves for each peptide. To quantitatively determine the peptides, an HPLC–MS/MS method was validated regarding the selectivity, linearity, precision, limit of detection (LOD) and quantification (LOQ). The transitions with higher signal intensities were selected as follows: HypSysI (519.8>498.2); HypSysII (595.5>494.6), HypSys III (518.3>394.2); Systemin (503.2>614.3); Potsys I (498.7>816.3); PotSys II (491.7>816.3); PepSys (395.8>392.2), and NishSys (506.3>515.3).

### Statistical Analysis

Statgraphics-plus software for Windows V.5 (Statistical Graphics Corp., MD, United States) was used to determine the statistical analysis by one-way analysis of variance (ANOVA) otherwise indicated in the figure legends. Means are shown with standard errors and their comparative was performed using Fisher’s least significant difference (LSD) at 99.5%. Graphs show the averages of one of the experiments. Each experiment contained a minimum of 6 plants per treatment and was repeated at least three times.

## Results

### Peptides From Different Plant Species Are Uptaken and Induce Resistance Against *Plectosphaerella cucumerina* in *Arabidopsis thaliana*

Plant peptides are involved in the majority of physiological plant processes. Most peptides that have been studied are peptides involved in plant growth and development. However, although there are some reports related to plant defense and induced resistance triggered by peptides, there remain large unexplored potentials of many peptides that may confer resistance against a wide range of pathogens and insects.

In a first attempt, we tested peptides for their potential activities in inducing plant resistance against fungal pathogens. To achieve this goal, we selected peptides from different plant species that were found to be involved in plant defense and performed screening bioassays of induced-resistance in the *Arabidopsis thaliana-Plectosphaerella cucumerina* pathosystem.

**FIGURE 1:**
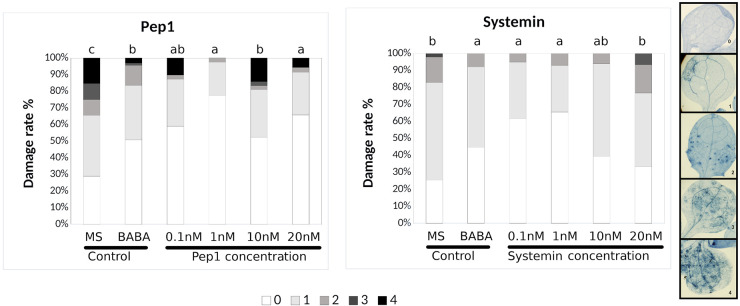
Pep1 and Systemin induced-resistance assays against *Plectosphaerella cucumerina* in Arabidopsis plants. Infection levels 5 days after inoculation quantified by a disease rating in trypan blue stained leaves, measured as a percentage of the infected leaf surface. Arabidopsis Col-0 plants were treated with increasing concentrations of Pep1 or Systemin (0.1, 1, 10, and 20 nM) 24 h before infection with 1 μl droplets of 5 × 10E3 spores/ml of *P. cucumerina* BMM. ß-amino butyric acid (BABA) at 1 ppm was used as a positive control. Colors mean % of diseased leaves in a scale (0 = healthy leaves; 1 = leaves with less than 25% of diseased surface; 2 = leaves with 25–50%; 3 = leaves with 50–75% of the diseased surface, 4 = leaves with more than 75% of the surface diseased). Different letters indicate statistically significant differences (ANOVA, Fisher’s Least Significant Difference (LSD) test; *P* < 0.05, *n* = 24). The experiment had 6 plants per treatment and was repeated at least three times with similar results.

**FIGURE 2:**
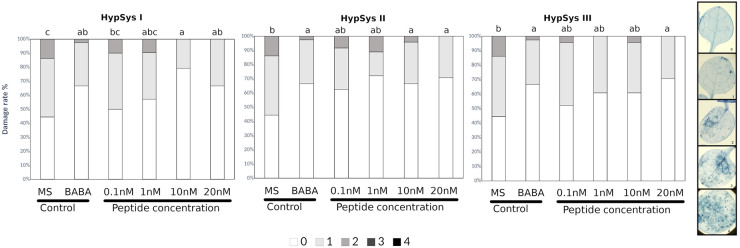
HypSys peptides induced-resistance assays against *Plectosphaerella cucumerina* in Arabidopsis plants. Infection levels 5 days after inoculation quantified by a disease rating in trypan blue stained leaves, measured as a percentage of the infected leaf surface. Arabidopsis Col-0 plants were treated with increasing concentrations of HypSysI, HypSysII, and HypSysII (0.1, 1, 10, and 20 nM) 24 h before infection with 1 μl droplets of 5 × 10E3 spores/ml of *P. cucumerina* BMM. ß-amino butyric acid (BABA) at 1 ppm was used as a positive control. Colors mean % of diseased leaves in a scale (0 = healthy leaves; 1 = leaves with less than 25% of diseased surface; 2 = leaves with 25–50%; 3 = leaves with 50–75% of the diseased surface, 4 = leaves with more than 75% of the surface diseased). Different letters indicate statistically significant differences (ANOVA, Fisher’s Least Significant Difference (LSD) test; *P* < 0.05, *n* = 24). The experiment had 6 plants per treatment and was repeated at least three times with similar results.

Pep1 from *Arabidopsis thaliana* (Huffaker et al., 2006; Yamaguchi et al., 2006; Klauser et al., 2015) and systemin from tomato were comparatively tested for induced resistance. As expected, Arabidopsis plants treated with AtPep1, which is known to function as an elicitor of plant defense in response to pathogens, exhibited significantly reduced severity of infection compared with water-treated controls at any of the concentrations tested (Figure 1 and Table 1). Systemin is an 18 aa peptide that has a function similar to that of AtPep1, although this peptide is mostly related to wounding and defense against insects in tomato (Fürstenberg-Hägg et al., 2013). Surprisingly, Systemin at very low concentrations (0.1 and 1 nM) was able to protect the plant against the necrotrophic fungus (Figure 1). Note that Pep1 and Systemin at the lowest concentrations (0.1 nM) protected plants to an extent similar to the protection conferred by □-amino butyric acid (BABA), a well-known inducer of resistance (Pastor et al., 2013). Subsequently, Systemins from other solanaceous species (potato, pepper, nightshade; Supplementary Figure S1; Constabel et al., 1998) were also tested. PepSys, NishSys and PotSysII were able to induce resistance at the same concentration as tomato Systemin. Note that all these peptides are produced in species that are taxonomically distant from *Arabidopsis thaliana* (Supplementary Figure S1). Moreover, we tested three short peptides from tomato, namely, HypSys I, HypSys II, and HypSys III, with functions in the defense against biotic stresses, although with a different sequence from Systemin. Arabidopsis plants were less sensitive to these peptides, although the plants treated with HypSysI and HypSysII at concentrations above 10 nM or with HypSys III at concentrations above 20 nM were also protected (Figure 2 and Table 1). These results suggest that Arabidopsis senses and responds to heterologous peptides.

**TABLE 1:** Peptides Induced-Resistance assays summary table.

**Peptide**	**Species of origin**	**0.1 nM**	**1 nM**	**10 nM**	**20 nM**
Pep1	Arabidopsis	+	+	+	+
Systemin	Tomato	+	+	*−*	*−*
PepSys	Pepper	+	+	*−*	*−*
NishSys	Nightshade	*−*	+	*−*	*−*
PotSys I	Potato	*−*	*−*	*−*	*−*
PotSys II	Potato	+	*−*	*−*	*−*
HypSys I	Tomato	*−*	*−*	+	+
HypSys II	Tomato	*−*	*−*	+	+
HypSys III	Tomato	*−*	*−*	*−*	+
AFP1	Radish	*−*	*−*	*−*	+
AFP2	Radish	*−*	*−*	*−*	+
Pep914	Soybean	*−*	*−*	*−*	*−*
Pep890	Soybean	*−*	*−*	*−*	*−*

**Peptides tested, their species of origin and the results obtained in the induce-resistance assays are shown in the table*. *(+) indicates effective plant protection and (−) indicates control levels of disease.**

**FIGURE 3:**
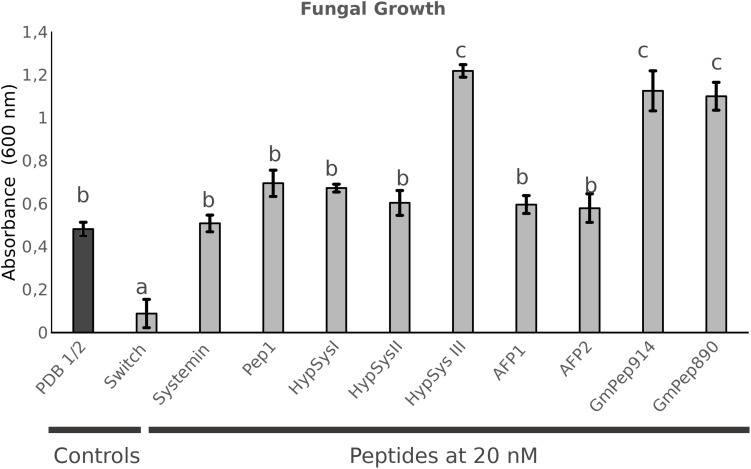
*In vitro* antifungal assays. *Plectosphaerella cucumerina* growth measured after 24 h growing in liquid medium containing each peptide at a concentration of 20 nM. Fungal growth was measured as the level of turbidity (absorbance 600 nM). A commercial fungicidal (Switch) was used as a positive control. Bars represent mean ± standard error (SD), *n* = 3. Different letters indicate statistically significant differences (ANOVA, Fisher’s Least Significant Difference (LSD) test; *P* < 0.05, *n* = 3).

The previous peptides were shown to function as DAMPs, stimulating the defensive responses following sensing of PAMPs. In addition, there are other peptides involved in defense display direct antimicrobial activity rather than activating signaling cascades. Two antimicrobial peptides (AMPs; AFP1, and AFP2) from radish that were described to be active against a broad spectrum of fungi were also tested for their ability to protect Arabidopsis against *P. cucumerina* (Terras et al., 1992; Supplementary Figure S2). AFP-treated plants showed significant levels of protection only at the highest concentration tested (20 nM) (Supplementary Figure S3). Finally, two short peptides from Soybean described as defense signals, GmPep914 and GmPep890, were also tested against *P. cucumerina*. These peptides lead to alkalinization of the medium and the activation of defense-related genes (Yamaguchi et al., 2011). None of these peptides succeeded in protecting Arabidopsis plants at any of the concentrations tested (Supplementary Figure S3). Interestingly, plants treated with 0.1 and 1 nM of GmPep91 are more susceptible to the fungus. This result correlates with the one shown in the antifungal assays (Figure 3) in which the fungal growth was higher in the presence of GmPep91. It is likely that the fungus is using this peptide as a source of amino acids.

It was previously shown that the T17A and P13AT17A truncated Systemin proteins were not functional at inducing resistance in tomato against fungal pathogens (Pearce et al., 1993; Xu et al., 2018). Furthermore, Sys-P13AT17A also failed to inhibit seedling root growth in Arabidopsis plants (Zhang et al., 2017). However, Sys-P13AT17A induced resistance in Arabidopsis against *P. cucumerina* at the same level as the natural tomato peptide (Supplementary Figure S4A). Alternatively, the functionality of the Arabidopsis peptide Pep1 was tested in tomato against *B. cinerea* and showed no significant protection (Supplementary Figure S4B).

Although it has been shown that some peptides and resistance inducers can produce direct cell death, in our experimental conditions, at all the concentrations used we did not observe any cell death in mock-infected plants following trypan blue staining. Therefore, we can assure that the cell death observed in our experiments is due to the infection.

Few methods for small peptides determination in solanaceous are found along the literature (Mucha et al., 2019). To further confirm the uptake and the presence of the non-self peptides that were able to induce resistance in Arabidopsis we developed a multi-residue analytical method based on the one described in Pastor et al. (2018). In this regard, a fast and accurate quantitative multi-residue method for the simultaneous determination of small peptides was developed. It was observed that the chromatographic standard peptides in plant complex matrices behaved very, similarly, to pure standard preparations, making it feasible to identify these peptides in any plant material following root treatments. With this method, we were able to detect and measure them in Arabidopsis plant samples after 24 h of the peptides’ treatment (Supplementary Figure S5).

### The Sequence Homology of Studied Peptides Is Not Linked to Their IR Activity

To determine whether the results in the screening assay of induced resistance could be explained by the phylogenetic proximity to *Arabidopsis thaliana* or sequence identity with the AtPep1, we performed multiple sequence alignment of the amino acid sequences of the peptides tested and built a phylogenetic tree based on the peptide sequences provided by the UniProt database.

By performing a Clustal Omega multiple sequence alignment, we discovered that the different peptides used in the screening have very low or nonexistent sequence homology with AtPep1 or with the other peptides tested (Supplementary Figure S6). Interestingly, the species that clade closer to Arabidopsis in the phylogenetic tree are those whose peptides either minimally protected (AFPs from radish) or failed to induce resistance (Peps from Soybean) against the fungus (Supplementary Figure S1). By comparison (Supplementary Figure S1 and Table 1), a correlation between the phylogenetic distance and effectively induced resistance against *P. cucumerina* in *Arabidopsis* was not observed.

In addition, we analyzed if the tested non-self peptides shared common motifs with AtPep1 that would account for their effectiveness in Arabidopsis. Using the Prosite database^3^, we found that Sys, PotSys1, PotSys2, PepSys, HypSys3, and Pep1 showed a serine protein kinase C phosphorylation site (red boxes in Supplementary Figure S6). Alternatively, AFP1 and AFP2 shared an N-myristoylation site (blue box). All these protein sites are patterns which have a high probability of occurrence, still they could not explain the different results obtained in the resistance induction assays (Supplementary Figure S6).

### The Studied Peptides Do Not Display Any Direct Antifungal Activity Against *P. cucumerina*

Because most peptides tested can protect Arabidopsis against the necrotrophic fungus, they likely exert either an induced resistance or a direct antimicrobial effect. To test this possibility, an *in vitro* assay to measure fungal growth in the presence of each peptide was performed. For the assay, we filled sterile 12-well plates with 3 ml of LB medium containing the peptide at the highest concentration (20 nM) to examine the toxic antimicrobial effect. Spores of *P. cucumerina* were added to each well, and fungal growth was measured 24 hpi by assessing the turbidity of the medium at 600 nm. A commercial fungicide (Switch^®^; Syngenta, 37.5% w/w cyprodinil and 25% w/w fludioxonil) at a concentration of 0.6 g.L^–1^ was used as a positive control (Figure 3). None of the peptides tested demonstrated antifungal activity against the necrotroph (Figure 3). Surprisingly, some of the peptides enhanced fungal growth, suggesting that the fungus may use the peptides as a source of amino acids.

These results suggest that the peptides induce resistance through the promotion of the plant immune system.

### Alterations in the Hormonal Imbalance May Contribute to Systemin-IR

For subsequent analysis, we focus on the tomato Systemin peptide since it was effective on inducing resistance at very low concentrations (Table 1). To further confirm Sys-IR using a different method for the infection quantification, fungal biomass related to the plant tissue was confirmed that it was significantly lower in plants treated with 0.1 nM Systemin (Supplementary Figure S7).

**FIGURE 4:**
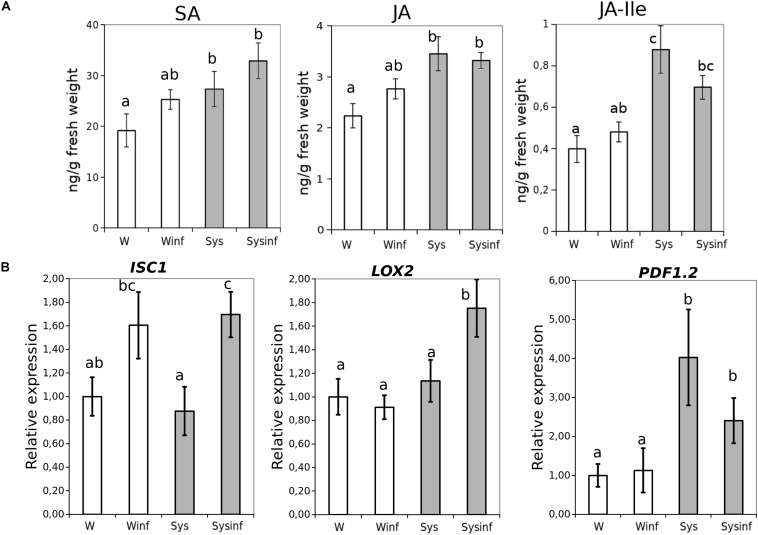
Systemin treatment impact in hormonal profiles. **(A)** Salicylic acid (SA), Jasmonic acid (JA) and JA-isoleucine (JA-ile) hormone quantitative levels (ng/g fresh weight) measured in Arabidopsis seedlings 48 h after *P. cucumerina* infection in control (W) control infected (W inf) 24 h Systemin-pretreated (Sys) and 24 h Systemin-pretreated infected (Sys inf) plants by targeted HPLC-MS analysis. **(B)** Quantitative reverse transcription-polymerase chain reaction analysis of *ICS1*, *LOX2*, and *PDF1.2* in seedlings 48 h after *P. cucumerina* infection in Water plants “W,” water infected plants “W inf,” 0.1 nM Systemin treated plants “Sys” and Sys infected plants “Sys inf.” Bars represent mean ± standard error (SD), *n* = 6. Different letters represent statistically significant differences. (ANOVA, Fisher’s Least Significant Difference (LSD) test; *P* < 0.05, *n* = 6).

In a first approach to understand the likely mechanisms of Systemin-IR in Arabidopsis, SA and JA as the main hormones regulating defense pathways were quantified (Figure 4A). In tomato, Systemin was shown to accumulate upon herbivory and was linked to JA-dependent responses (Sun et al., 2011; Fürstenberg-Hägg et al., 2013). In Arabidopsis, 0.1 nM Systemin treatments triggered an increase in SA, JA and JA-Ile in the absence of infection compared to water-treated plants. In contrast, following infection, the hormonal levels in Arabidopsis plants treated with Systemin remained similar to the levels before the infection. These observations suggest that SA- and JA-dependent pathways may contribute to Systemin-IR, however, the hormonal changes triggered by Systemin take place independently of the infection.

To complement the previous observations on the hormonal imbalances, we performed an analysis of *ICS1, LOX2*, and *PDF1.2* gene expression (Figure 4B). The JA-biosynthesis gene *LOX2* was boosted by systemin in the presence of infection displaying a priming profile (Mauch-Mani et al., 2017), whereas PDF1.2 gene expression was triggered by the treatment independently of the infection. *ICS1* expression levels increased due to the infection being significantly higher only in plants treated with Systemin.

**FIGURE 5:**
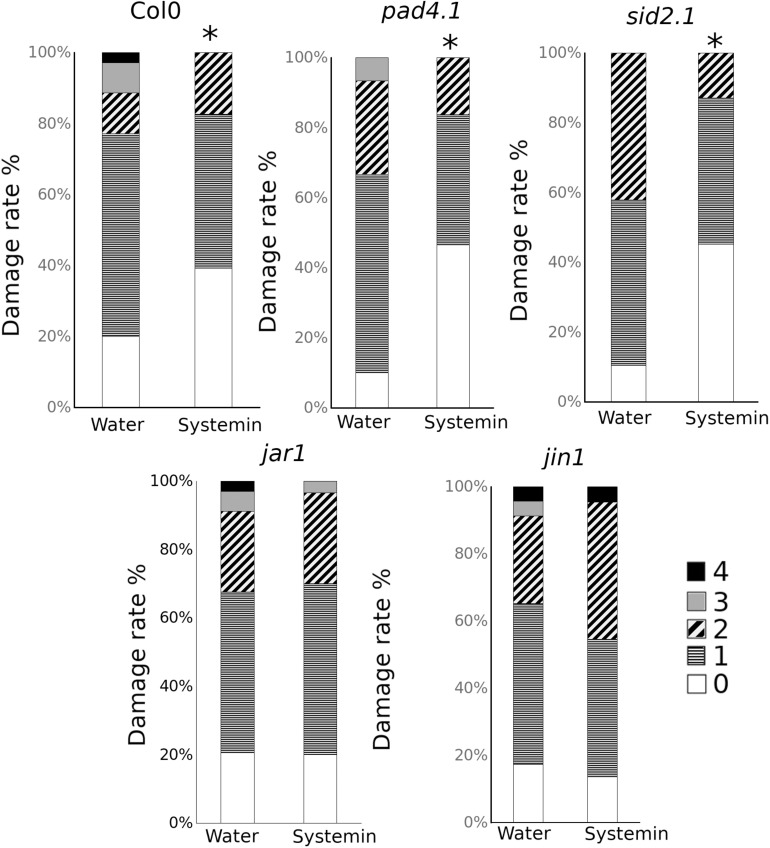
Sys-IR assays in mutants impaired in the SA and JA-related pathways. Col-0, *pad4.1*, *sid2.1*, *jar1*, and jin1 plants were challenged with 1 μl droplets of 5 × 10E3 spores/ml of *P. cucumerina* BMM 24 h after treatment with 0.1 nM Systemin. Infection levels were quantified 5 days after inoculation by a disease rating in trypan blue stained leaves, measured as a percentage of the infected leaf surface. Colors mean % of diseased leaves in a scale (0 = healthy leaves; 1 = leaves with less than 25% of diseased surface; 2 = leaves with 25–50%; 3 = leaves with 50–75% of the diseased surface, 4 = leaves with more than 75% of the surface diseased). Asterisks mean statistical significant differences; *T*-test; *P* < 0.05, *n* = 12). The experiment had 12 plants per treatment and was repeated at least three times with similar results.

To be more confident about the role of both hormonal pathways, mutants impaired in the SA and JA-related pathways were treated and infected (Figure 5). Interestingly, only those mutants altered in the JA responses were impaired in the Systemin-IR, while the SA-related *pad 4.1* and *sid2.1* mutants were protected by the peptide.

Based on these results, although SA is induced by Systemin treatments, the gene expression and the mutant analysis suggest that, like in tomato, JA-dependent responses may regulate Systemin-IR in Arabidopsis. However, JA functions in Systemin-IR may likely happen coordinately with other yet unknown mechanisms to contribute to the observed induced resistance phenotype.

### Systemin Enhances PTI Responses in Arabidopsis

**FIGURE 6:**
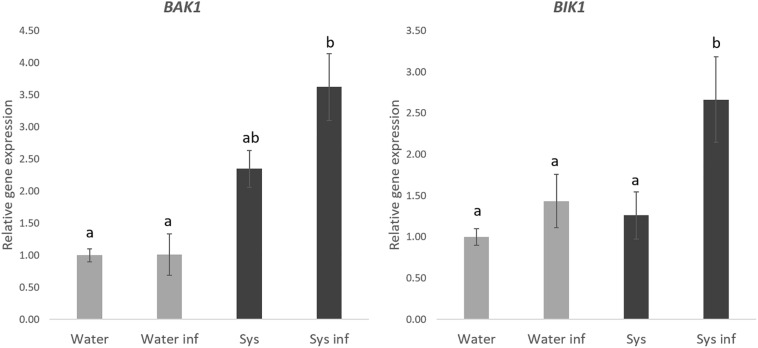
Systemin treatment impact in ***BAK1*** and ***BIK1*** gene expression. Quantitative reverse transcription-polymerase chain reaction (qPCR)analysis of *BAK1* and *BIK1* in seedlings 48 h after *P. cucumerina* infection in normal water plants “W,” water infected plants “W inf,” 0.1 nM Systemin treated “Sys” and Sys infected plants “Sys inf” plants. Bars represent mean ± standard error (SD), *n* = 6. Different letters represent statistically significant differences (ANOVA, Fisher’s Least Significant Difference (LSD) test; *P* < 0.05, *n* = 6).

To gain knowledge on the perception and signaling of tomato Systemin in Arabidopsis we analyzed some well-known PTI responses. On the one hand, we measured the expression of the *BAK1* and *BIK1* membrane receptors as PTI markers in Arabidopsis plants treated with systemin and challenged with spores of *P. cucumerina* (Figure 6). None of the tested genes was directly induced by systemin treatments. However, both PTI markers were strongly upregulated in treated plants after infection (Figure 6), showing a typical priming profile.

**FIGURE 7:**
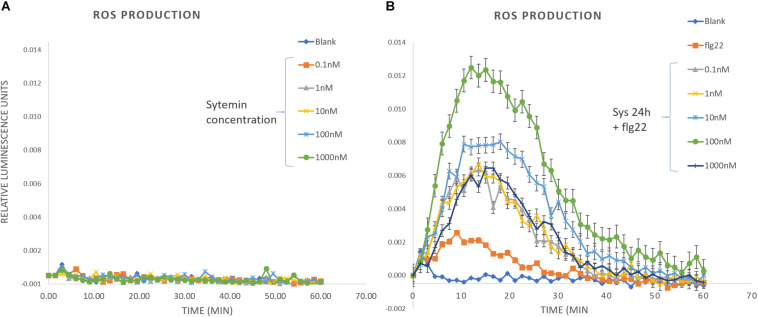
ROS production in response to Systemin and PAMP challenge. H_2_O_2_ production was measured during 1 h in leaf disks after elicitation with **(A)** Systemin at different concentrations and **(B)** 100 nM flg22 in leaf disks that were pre-treated for 24 h with different concentrations of Systemin. Luminescence was expressed in Relative Luminescence Units. Slopes represent the means of each time-point ± standard error (SD), *n* = 8.

On the other hand, we measured ROS production induced by Systemin and a PAMP challenge after 24 h systemin treatment (Figure 7). A wide range of Systemin concentrations was used (0.1, 1, 10, 100, and 1000 nM). Systemin treatments in the absence of a PAMP did not induce the production of H_2_O_2_ (Figure 7 and Supplementary Figure S8) but ROS production was significantly induced when plants that were treated with Systemin 24 h before and challenged with flg22 (Figure 7). The induction was higher with increasing concentrations of Systemin showing a maximum threshold (100 nM). When Systemin was applied at higher concentrations the ROS accumulation decayed to levels similar to 0.1 nM os Systemin. This result shows a dose-threshold response of Arabidopsis to Systemin, resembling the protection pattern that we observed in the IR assays (Figure 1 and Table 1). The results commented above suggest that Arabidopsis perceives tomato Systemin but in a non-canonnical perception unlike classical DAMPs such as Pep1. To further study this hypothesis we confirmed that the mutant pepr1 displays a wild-type phenotype of Sys-IR (Supplementary Figure S9), hence this reinfoced a PEPR1-independent function of systemin.

## Discussion

The understanding of small peptides as signaling molecules in plants has grown significantly in the last few years. In the present study, the role of Arabidopsis self and non-self peptides in inducing resistance against *P. cucumerina* has been analyzed. Reasonably, self-peptides are active in protecting Arabidopsis, but surprisingly, other heterologous peptides, such as Systemins from Solanum species, protect Arabidopsis in the nanomolar range. Besides, other peptides from phylogenetically distant plant species are also active in defense, although to a different extent.

Alternatively, most knowledge of small peptides functioning throughout the plant physiology has been generated by studying the gene expression of their respective propeptides. However, the post-translational processing of these propeptides is tightly regulated, which makes the analytical characterization and quantification of the active peptides essential. For this reason, we have generated a multi-residue UPLC coupled to mass spectrometry method for the simultaneous analysis of small plant peptides (15–20 amino acids).

Small peptides were shown to participate in plant defense as amplifiers of PAMP sensing; therefore, they were suggested to function as DAMPs, which are also known as phytocytokines (Gust et al., 2017). For instance, PIPs from Arabidopsis were shown to amplify flg22 responses and resistance to *PstDC3000* (Hou et al., 2014), and similarly, elf18 responses increased upon co-treatment with RALF17 (Stegmann et al., 2017). Previous studies described the functionality of the Arabidopsis endogenous peptide Pep1 in the defense against fungal pathogens, such as *B. cinerea* (Liu et al., 2013). In the current study, Pep1 exogenously applied in a range from 0.1 to 20 nM was found to protect plants against *P. cucumerina*. Pep1, at the concentrations tested, was as functional as the well-known priming agent □-amino butyric acid (BABA). In parallel, a screening of non-self peptides for induced resistance against the necrotroph was performed. The screening included peptides from other Brassicaceae, such as AFP1 and 2 (Terras et al., 1992), Solanaceae, such as Systemin, PepSys, NishSys, PotSysI and II (Constabel et al., 1998), HypSys I, II, and III (Pearce and Ryan, 2003), and Fabaceae, such as Pep914 and 890 (Yamaguchi et al., 2011). Unexpectedly, the solanum peptides were the most effective in protecting Arabidopsis. Systemin-induced resistance from tomato and pepper and PEP1-IR were as strong as that induced by BABA-IR and Pep1-IR at the very low concentrations of 0.1 and 1 nM. In contrast, a Systemin from potato (PotSysI) and peptides from soybean (Pep914 and 890) did not induce resistance at the concentrations studied. HypSys I, II, and III as well as AFP1 and 2 demonstrated protection only at the highest concentrations. These observations suggest that either Arabidopsis has specific receptor(s) for heterologous plant peptides, which is rather unlikely, or that other yet unknown receptors may bind nonspecifically other small peptides. Further research is needed to clarify this hypothesis.

Because induced resistance was observed, a double analysis of the peptides was performed. The likely link between phylogenetic proximity of the plant species that produce the peptides and the effectiveness inducing resistance was studied. The phylogenetic distance of radish is closer to Arabidopsis compared with tomato, pepper or soybean, although systemins from tomato and pepper were the most effective. Hence, the protection conferred by the tested peptides may not be related to the phylogenetic proximity of the plant species. Second, the sequence homology and the motifs contained in the peptides were also studied. Any of these biochemical properties were linked to higher efficiency in protection. In fact, Pep1 from Arabidopsis shares higher sequence homology with AFPs and Pep from soybean, while Systemin, PepSys and PotSysI and II share very high sequence homology. Note that Systemin and PepSys treatments induced strongly Arabidopsis resistance against the fungus, while PotSysI treatment was ineffective. Alternatively, the only motif shared by these small peptides was a phosphorylation site that was present in Systemin, PepSys, Pep1, PotSys1, PotSys2, and HypSys3. Therefore, neither a conserved sequence nor specific motifs can explain the differential function in Arabidopsis protection.

To fully exclude the possibility that these peptides protect Arabidopsis by inhibiting *P. cucumerina* growth or germination, the *in vitro* antimicrobial effect of all peptides at the highest concentration was tested. None of the small peptides inhibited fungal growth, although surprisingly some of them promoted mycelium expansion, such as HypSys III from tomato and Pep914 and 890 from soybean. These peptides may function as additional nutritional sources for the fungus, which would explain its enhanced growth. Especially surprising was the absence of an antimicrobial effect of the antifungal peptides AFP1 and 2, since their inhibitory properties against several fungi, including the necrotroph *B. cinerea*, have been previously shown, although at concentrations higher than those used in our tests (Terras et al., 1992*;* De Lucca et al., 1999; Thevissen et al., 2012*)*. Regarding the remaining peptides, any of them either promoted or reduced fungal growth, which suggest they protect Arabidopsis through activation of the plant immunity.

Under our experimental conditions, Pep1 treatments protected Arabidopsis plant at any of the concentrations tested (0.1–20 nM). Nevertheless, Systemin treatments significantly protected Arabidopsis at the very low doses of 0.1 and 1 nM, but it was not active at the higher concentrations.

This mode of action has been previously reported for some well-known resistance inducers and phytohormones. BABA shows a threshold of protection against *Phytophthora infestans* between 1 and 10 mM while 0.1 and 20 mM are less effective (Floryszak-Wieczorek et al., 2015). Moreover, BABA-induced callose accumulation in response to PAMPs has also a maximum in the range of 1–5 ppm, while decays at higher concentrations (Pastor et al., 2013). Similarly, BTH was shown to protect better a low doses triggering PAL and inducing coumarin accumulation (Katz et al., 1998). Regarding phytohormones, as an example, brassinosteroid showed maximum threshold on promoting root elongation, while they trigger root elongation at low doses (0.05–0.1 nM) they fail above 1 nM (Müssig et al., 2003). Therefore we can assume that Systemin-IR in arabidopsis acts in a dose-threshold manner, what was also confirmed by the ROS assays.

There are reports of enhanced resistance of transgenic Arabidopsis plants overexpressing the Prosystemin gene (Zhang et al., 2017). The overexpression of Prosystemin has a strong impact on the Arabidopsis transcriptome with upregulation of stress-related genes. Prosystemin is a 200 amino acid peptide that is processed in tomato by phytaspases. Subsequently, leucine aminopeptidase A removes the terminal Leu, releasing the active form of systemin (Beloshistov et al., 2017). Despite the functionality of overexpression of prosystemin in Arabidopsis, it is still unknown whether the propeptide is active by itself or whether other Arabidopsis phytaspases and a LapA-like protein can process Prosystemin. In the present experiments, it was shown that not only Systemin but also its truncated form Sys-P13AT17A (Pearce et al., 1993) are sensed by Arabidopsis. This result suggests that a core of amino acids in the peptide may be responsible for the non-specific perception and downstream signaling in Arabidopsis since the truncated forms are entirely impaired in inducing resistance in tomato (Pearce et al., 1993; Xu et al., 2018).

Conversely, Pep1 treatments did not protect tomato plants against *B. cinerea*. Thus, it appears that tomato very specifically senses Systemin but not Pep1, while Arabidopsis can sense Pep1 though its known receptors (PEPR1 and 2) and Systemin through an unknown mechanism. In this regard, not only Systemin but also several other tested peptides, such as PepSys, NighSys, HypSys I, II, and III, can induce resistance in Arabidopsis, although at higher concentrations. This finding reinforces the hypothesis that Arabidopsis may have alternative non-specific receptors for non-self peptides. It is tempting to hypothesize that extracellular peptides, as it has been shown for DNA, ATP or oxylipins released form the membrane may function as danger signals, although not all peptides exert the same activity.

As a first approach to decipher mechanisms underlying Sys-IR, a hormonal analysis showed that SA- and JA-related signaling could be involved. Despite their antagonism, both SA and JA increased following Systemin treatments in Arabidopsis. The active hormone JA-Ile was also triggered following Systemin treatments. Accordingly, several hormone-related genes, such as *LOX2* and *PDF1.2* from the JA-dependent pathway, were also induced by Systemin treatments. The hormone induction and the gene expression have consistent behavior in the activation of both pathways in Systemin-treated plants upon infection, indicating that a more complex regulation of defenses may occur following Systemin sensing that indeed has an impact on hormonal signaling. Note that the PEPR pathway co-activates SA- and JA/ET- mediated immune branches in Arabidopsis (Ross et al., 2014). Despite the induction of SA levels after Systemin treatments, the mutant analysis showed that SA-impaired mutants were fully protected suggesting that JA-dependent responses are behind Sys-IR in Arabidopsis. Similarly, Systemin treatments have been shown to trigger JA-dependent responses in tomato (Ryan, 2000; Sun et al., 2011; Fürstenberg-Hägg et al., 2013) and involve the upstream oxylipin pathway following herbivory. Thus, the JA induction following Systemin treatments appears to be a conserved molecular response in Arabidopsis and tomato.

To understand Systemin perception in Arabidopsis we analyzed both *BAK1* and *BIK1* gene expression and the generation of ROS. Following Systemin treatment any of the studied markers were directly induced. However, following *P. cucumerina* infection both transcripts increased significantly and additionally flg22 application in Systemin-treated plants induced strong increases in ROS production. To strengthen these observations, we confirmed that Sys-IR is functional in the mutant *pepr1*, hence PEPR1-independent. Note that it was reported previously that systemin effects on root architecture in Arabidopsis is also PEPR1-independent (Zhang et al., 2017). This suggests that Arabidopsis senses Systemin although it is inducing a non-canonical function compared with endogenous peptidic DAMPs such as Pep1/2 that directly induce responses. Although Systemin clearly amplifies PAMP/pathogen response, it is likely that the low doses used do not trigger direct responses resembling priming defense as it has been previously suggested for other priming stimuli (Mauch-Mani et al., 2017; Wilkinson et al., 2019).

Much of the understanding of the function of peptides in plant immunity has been based on propeptide gene expression. In very few cases, the processing of these propeptides, the final receptors and signaling cascades have only been recently discovered (Yamaguchi et al., 2006; Hou et al., 2014; Wang et al., 2018; Xu et al., 2018). Following the propeptide translation, proteolytic processing is involved in the cleavage and release of the active peptide from a larger precursor. Non-self peptides should not be specifically processed in Arabidopsis, since they are not naturally present, although it could be possible that they can be processed by other non-specific phytaspases or peptidases that are ubiquitous among plants. Using a multi-residue chromatographic method we have confirmed the uptake and systemic transport of the heterologous peptides in Arabidopsis.

## Conclusion

In conclusion, Systemin and other related peptides that are not produced in Arabidopsis can induce resistance against *P. cucumerina*, triggering protection at very low doses and to a comparable extent as the protection provided by BABA, which indicated that Arabidopsis can sense non-self peptides from phylogenetically distant plant species that are not related in structure or sequence. Furthermore, we show evidence that the JA-dependent signaling mediates Systemin-Induced Resistance that amplifies PAMP receptor expression and ROS production in the presence of a challenge. Pre-challenge induction may prepare the plant for subsequent exposure. These findings open future research to decipher the mechanisms underlying Sys-IR in Arabidopsis.

## Data Availability Statement

All datasets generated for this study are included in the article/Supplementary Material.

## Author Contributions

JP-F performed most bioassays of IR and peptide treatments. JG and VP developed the LC-MS methods and contributed to the writing of results and methods. PS-B contributed to writing, interpretation, motif, and peptide sequence analysis. NS performed assays with tomato and the mutant screenings. MC contributed to PCR analysis. VF contributed to writing, supervised the research, designed experiments, and performed hormonal analysis.

## Conflict of Interest

The authors declare that the research was conducted in the absence of any commercial or financial relationships that could be construed as a potential conflict of interest.

## Footnotes

1
www.sigmaaldrich.com

2
https://www.biomatik.com/

3
http://wwwuser.cnb.csic.es/~pazos/cam97/

## Supplementary Material

FIGURE S1 Systemins from Solanaceous species induced-resistance assays against *Plectosphaerella cucumerina* in Arabidopsis plants. Infection levels 5 days after inoculation quantified by a disease rating in trypan blue stained leaves, measured as a percentage of the infected leaf surface. Arabidopsis Col-0 plants were treated with increasing concentrations of PotSys (potato systemin), PepSys (pepper systemin), NishSys (nightshade systemin) (0.1, 1, 10, and 20 nM) 24 h before infection with 1 μl droplets of 5 × 10E3 spores/ml of *P. cucumerina* BMM. ß-amino butyric acid (BABA) at 1 ppm was used as a positive control. Colors mean % of diseased leaves in a scale (0 = healthy leaves; 1 = leaves with less than 25% of diseased surface; 2 = leaves with 25–50%; 3 = leaves with 50–75% of the diseased surface, 4 = leaves with more than 75% of the surface diseased). Different letters indicate statistically significant differences (ANOVA, Fisher’s Least Significant Difference (LSD) test; *P* < 0.05 *n* = 6). The experiment had 6 plants per treatment and was repeated at least three times with similar results.

FIGURE S2 Antimicrobial peptides from radish induced-resistance assays against *Plectosphaerella cucumerina* in Arabidopsis plants. Infection levels 5 days after inoculation quantified by a disease rating in trypan blue stained leaves, measured as a percentage of the infected leaf surface. Arabidopsis Col-0 plants were treated with increasing concentrations of AFP1 and AFP2 (0.1, 1, 10, and 20 nM) 24 h before infection with 6 μl droplets of 5 × 10E3 spores/ml of *P. cucumerina* BMM. ß-amino butyric acid (BABA) at 1 ppm was used as a positive control. Colors mean % of diseased leaves in a scale (0 = healthy leaves; 1 = leaves with less than 25% of diseased surface; 2 = leaves with 25–50%; 3 = leaves with 50–75% of the diseased surface, 4 = leaves with more than 75% of the surface diseased). Different letters indicate statistically significant differences (ANOVA, Fisher’s Least Significant Difference (LSD) test; *P* < 0.05, *n* = 6). The experiment had 6 plants per treatment and was repeated at least three times with similar results.

FIGURE S3 Soybean peptides induced-resistance assays against *Plectosphaerella cucumerina* in Arabidopsis plants. Infection levels 5 days after inoculation quantified by a disease rating in trypan blue stained leaves, measured as a percentage of the infected leaf surface. Arabidopsis Col-0 plants were treated with increasing concentrations of GmPep914 and GmPep890 (0.1, 1, 10, and 20 nM) 24 h before infection with 1 μl droplets of 5 × 10^3^ spores/ml of *P. cucumerina* BMM. Colors mean % of diseased leaves in a scale (0 = healthy leaves; 1 = leaves with less than 25% of diseased surface; 2 = leaves with 25–50%; 3 = leaves with 50–75% of the diseased surface, 4 = leaves with more than 75% of the surface diseased). Different letters indicate statistically significant differences (ANOVA, Fisher’s Least Significant Difference (LSD) test; *P* < 0.05, *n* = 6). The experiment had 6 plants per treatment and was repeated at least three times with similar results.

FIGURE S4 Induced- Resistance assays of Sys-P13AT17A in Arabidopsis and AtPep1 in tomato. Infection levels of Arabidopsis Col-0 plants treated with 0.1 nM of truncated Systemin (Sys-P13AT17A) **(A)** and tomato wild-type plants treated with increasing concentrations of AtPep1 (0.1, 1, 10, and 20 nM) **(B)** 24 h before infection. Infection was quantified 5 days after inoculation with 1 μl droplets of 5 × 10^3^ spores/ml of *P. cucumerina* BMM by a disease rating in trypan blue stained leaves, measured as a percentage of the infected leaf surface. Colors mean % of diseased leaves in a scale (0 = healthy leaves; 1 = leaves with less than 25% of diseased surface; 2 = leaves with 25–50%; 3 = leaves with 50–75% of the diseased surface, 4 = leaves with more than 75% of the surface diseased). Different letters indicate statistically significant differences (ANOVA, Fisher’s Least Significant Difference (LSD) test; *P* < 0.05, *n* = 6). The experiment had 6 plants per treatment and was repeated at least three times with similar results.

FIGURE S5 Peptides measured by HPLC-MS *in planta.* **(A)** total ion current (TIC) in ESI (+) of a mix of peptide standards and **(B)** HPLC–MS/MS chromatograms of specific transitions for each peptide of study detected in Arabidopsis plants 24 h after peptide treatment. Aliquots of 20 μl of a standard mix of 300 μl.L^–1^ were injected into the LC-MS system through a reversed column, at a flow rate of 0.3 ml min^–1^. After data recording, chromatograms were generated using the Maslynx 4.1 (Waters) software.

FIGURE S6 Peptides phylogenetic tree and multiple alignment based on their amino acid sequence. Phylogenetic tree and multiple alignment were performed using the Clustal Omega multiple alignment of the EMBL-EBI online tool (https://www.ebi.ac.uk/Tools/msa/clustalo/) using the peptides amino acid sequence provided by the Uniprot database. Numbers on the right indicate peptides’ length (number of aminoacids). Highlighted in boxes are the motifs found in each peptide using the Prosite Database (http://wwwuser.cnb.csic. es/∼pazos/cam97/). Red boxes indicate the Serine Protein Kinase C phosphorylation sites, blue box indicate N-myristoylation sites.

FIGURE S7 *P. cucumerina* Infection quantification by measuring fungal biomass. A ratio of *PcTUBULIN* relative to *AtUBIQUITIN21* was calculated after performing a qPCR from gDNA of Arabidopsis infected plant samples 48 h after pathogen inoculation in watered plants and plants treated with 0.1 nM systemin 24 h before inoculation of *P. cucumerina.* Bars represent mean ± standard error (SD), *n* = 6. Asterisks mean statistical significant differences; *T*-test; *P* < 0.05, *n* = 6.

FIGURE S8 ROS production areas in response to Systemin and PAMP challenge. H_2_O_2_ production was measured during 1 h in leaf disks after elicitation with Systemin at different concentrations and with 100 nM flg22 in leaf disks that were pre-treated for 24 h with different concentrations of Systemin. Luminescence was expressed in Relative Luminescence Units. Bars represent means of peak areas ± standard error (SD), *n* = 8. Different letters represent statistically significant differences. (ANOVA, Fisher’s Least Significant Difference (LSD) test; *P* < 0.05, *n* = 8).

FIGURE S9 Sys-IR assays in the *pepr1* mutant. Col-0 and *pepr1* plants were challenged with 1 μl droplets of 5 × 10E3 spores/ml of *P. cucumerina* BMM 24 h after treatment with 0.1 nM Systemin. Infection levels were quantified 5 days after inoculation by a disease rating in trypan blue stained leaves, measured as a percentage of the infected leaf surface. Colors mean % of diseased leaves in a scale (0 = healthy leaves; 1 = leaves with less than 25% of diseased surface; 2 = leaves with 25–50%; 3 = leaves with 50–75% of the diseased surface, 4 = leaves with more than 75% of the surface diseased). Asterisks mean statistical significant differences; *T*-test; *P* < 0.05, *n* = 12). The experiment had 12 plants per treatment and was repeated at least three times with similar results.

TABLE S1 Primers used for the qPCR analysis of gene expression and *Plectosphaerella cucumerina* quantification.
