# Corrigendum: Arabidopsis plants sense non-self peptides to promote resistance against *Plectosphaerella cucumerina*

**DOI:** 10.3389/fpls.2024.1449013

**Published:** 2024-07-04

**Authors:** Julia Pastor-Fernández, Jordi Gamir, Victoria Pastor, Paloma Sanchez-Bel, Neus Sanmartín, Miguel Cerezo, Víctor Flors

**Affiliations:** Metabolic Integration and Cell Signaling Laboratory, Plant Physiology Section, Unidad Asociada al Consejo Superior de Investigaciones Científicas (EEZ-CSIC)-Department of Ciencias Agrarias y del Medio Natural, Universitat Jaume I, Castellón, Spain

**Keywords:** systemin, induced resistance, Arabidopsis, LC-MS, *Plectoshaerella cucumerina*

In the published article, in **Figures 1** and **2** as well as Supplementary Figures 1, 2 and 3 the picture legend on the right side of the figures shows an example of the % of damage in the leaves. We have updated the independent scale of diseased leaves for **Figures 1** and **2** and we have deleted the scale legend in the Supplementary Figures 1, 2 and 3. The corrected legend appears now in the updated **Figures 1** and **2** below, and in the Supplementary Material in the original files.

**Figure 1:**
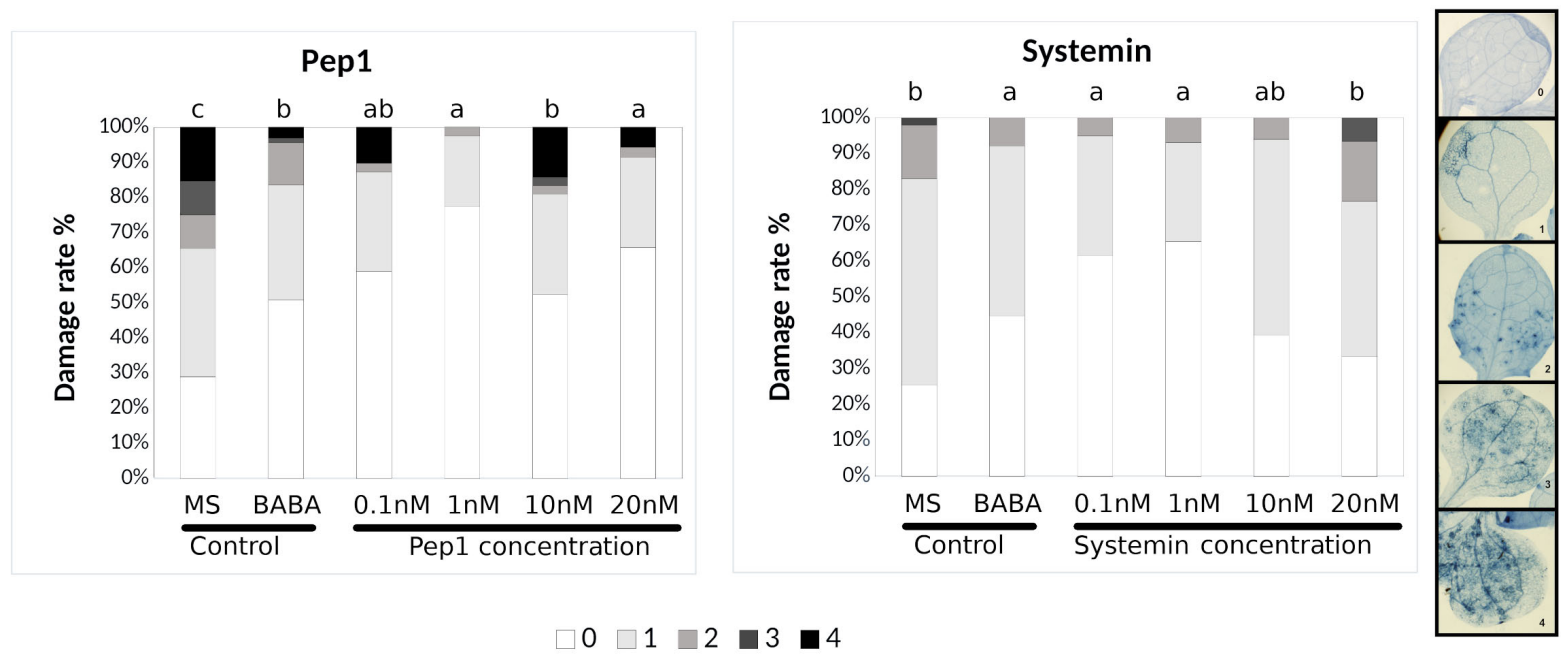
Pep1 and Systemin induced-resistance assays against *Plectosphaerella cucumerina* in Arabidopsis plants. Infection levels 5 days after inoculation quantified by a disease rating in trypan blue stained leaves, measured as a percentage of the infected leaf surface. Arabidopsis Col-0 plants were treated with increasing concentrations of Pep1 or Systemin (0.1, 1, 10, and 20 nM) 24 h before infection with 1 μl droplets of 5 × 10E3 spores/ml of *P. cucumerina* BMM. ß-amino butyric acid (BABA) at 1 ppm was used as a positive control. Colors mean % of diseased leaves in a scale (0 = healthy leaves; 1 = leaves with less than 25% of diseased surface; 2 = leaves with 25–50%; 3 = leaves with 50–75% of the diseased surface, 4 = leaves with more than 75% of the surface diseased). Different letters indicate statistically significant differences (ANOVA, Fisher’s Least Significant Difference (LSD) test; *P* < 0.05, n = 24). The experiment had 6 plants per treatment and was repeated at least three times with similar results.

**Figure 2:**
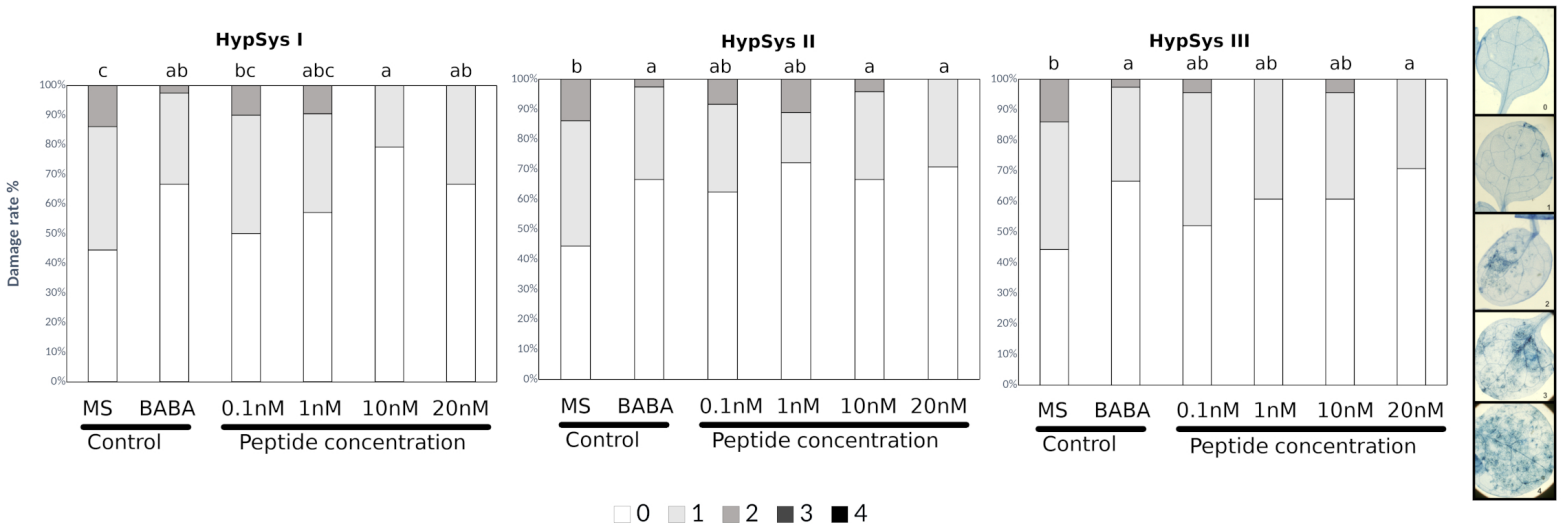
HypSys peptides induced-resistance assays against *Plectosphaerella cucumerina* in Arabidopsis plants. Infection levels 5 days after inoculation quantified by a disease rating in trypan blue stained leaves, measured as a percentage of the infected leaf surface. Arabidopsis Col-0 plants were treated with increasing concentrations of HypSysI, HypSysII, and HypSysII (0.1, 1, 10, and 20 nM) 24 h before infection with 1 μl droplets of 5 × 10E3 spores/ml of *P. cucumerina* BMM. ß-amino butyric acid (BABA) at 1 ppm was used as a positive control. Colors mean % of diseased leaves in a scale (0 = healthy leaves; 1 = leaves with less than 25% of diseased surface; 2 = leaves with 25–50%; 3 = leaves with 50–75% of the diseased surface, 4 = leaves with more than 75% of the surface diseased). Different letters indicate statistically significant differences (ANOVA, Fisher’s Least Significant Difference (LSD) test; *P* < 0.05, n = 24). The experiment had 6 plants per treatment and was repeated at least three times with similar results.

The authors apologize for this error and state that this does not change the scientific conclusions of the article in any way. The original article has been updated.

