# Supplementary figures and images for: Arabidopsis Plants Sense Non-self Peptides to Promote Resistance Against Plectosphaerella cucumerina
Source: Front Plant Sci. 2020 May 8;11:529. doi: 10.3389/fpls.2020.00529 (PMC7225342; doi:10.3389/fpls.2020.00529)

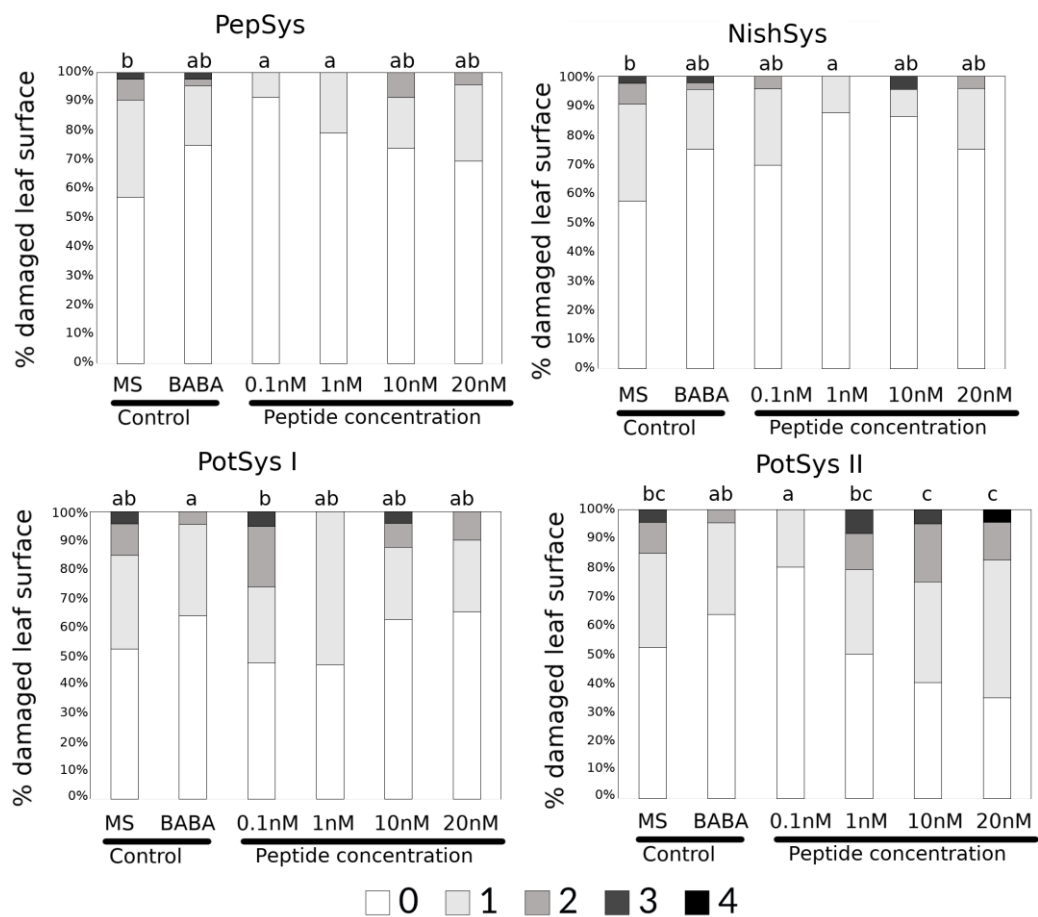

Figure S1

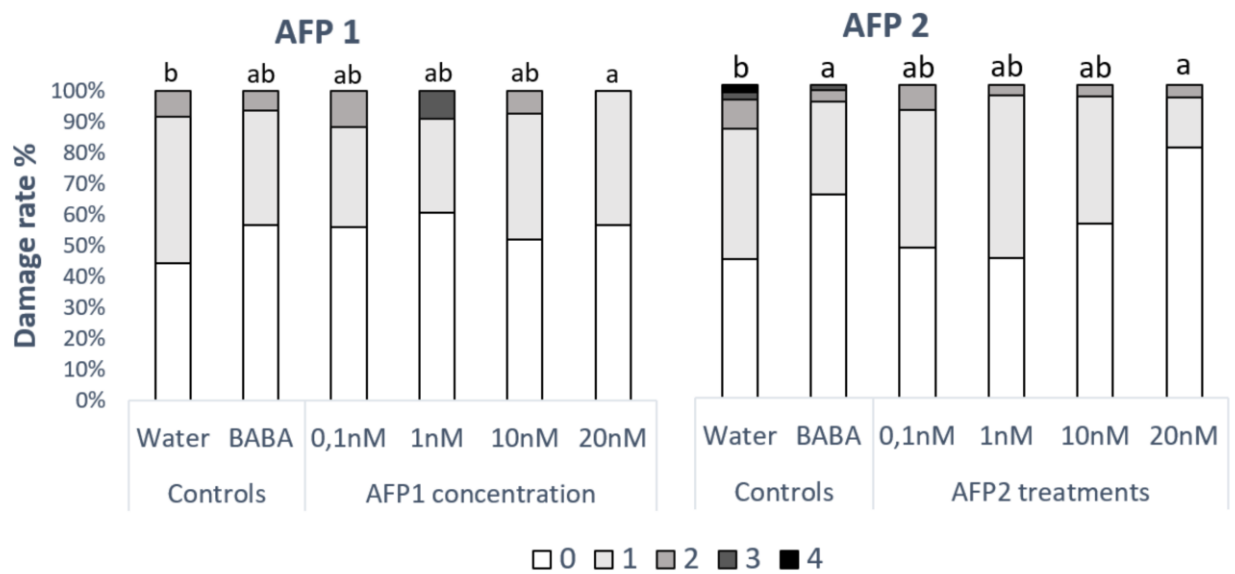

Figure S2

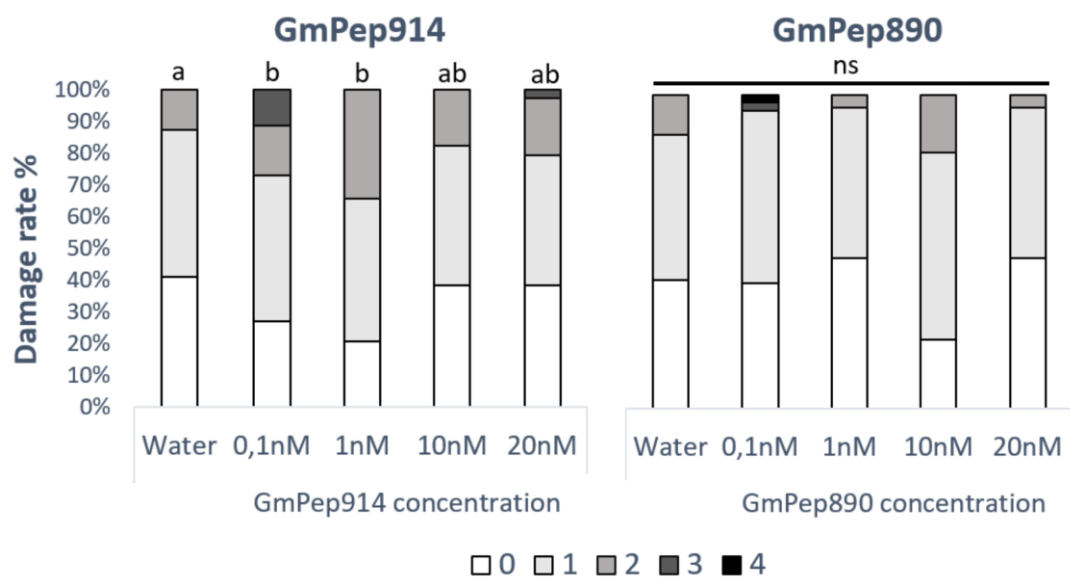

Figure S3

Supplement: FIGURE S1 — Systemins from Solanaceous species induced-resistance assays against Plectosphaerella cucumerina in Arabidopsis plants. Infection levels 5 days after inoculation quantified by a disease rating in trypan blue stained leaves, measured as a percentage of the infected leaf surface. Arabidopsis Col-0 plants were treated with increasing concentrations of PotSys (potato systemin), PepSys (pepper systemin), NishSys (nightshade systemin) (0.1, 1, 10, and 20 nM) 24 h before infection with 1 μl droplets of 5 × 10E3 spores/ml of P. cucumerina BMM. ß-amino butyric acid (BABA) at 1 ppm was used as a positive control. Colors mean % of diseased leaves in a scale (0 = healthy leaves; 1 = leaves with less than 25% of diseased surface; 2 = leaves with 25–50%; 3 = leaves with 50–75% of the diseased surface, 4 = leaves with more than 75% of the surface diseased). Different letters indicate statistically significant differences (ANOVA, Fisher’s Least Significant Difference (LSD) test; P < 0.05 n = 6). The experiment had 6 plants per treatment and was repeated at least three times with similar results. [file Data_Sheet_1.pdf]

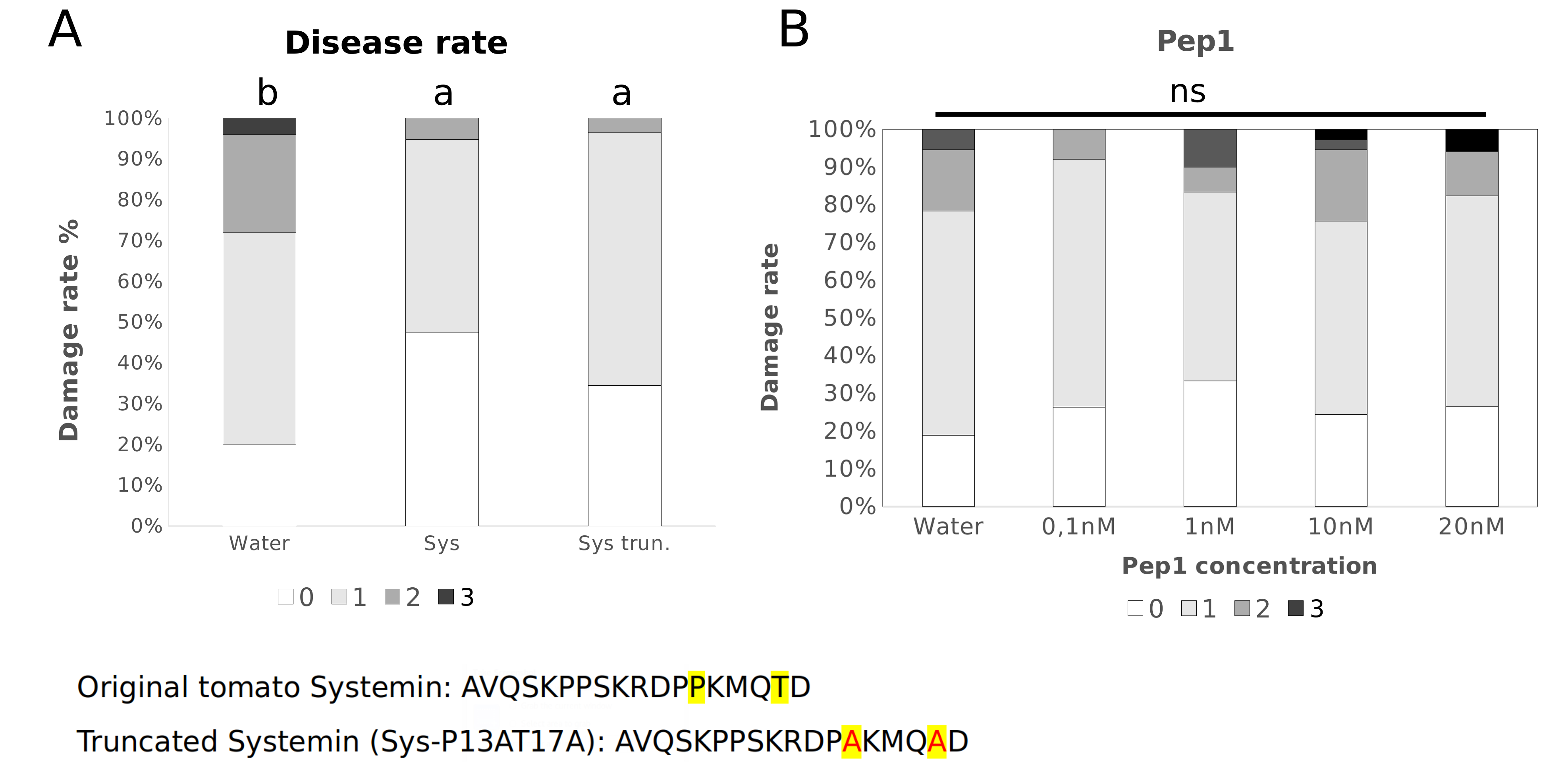

Supplement: FIGURE S4 — Induced- Resistance assays of Sys-P13AT17A in Arabidopsis and AtPep1 in tomato. Infection levels of Arabidopsis Col-0 plants treated with 0.1 nM of truncated Systemin (Sys-P13AT17A) (A) and tomato wild-type plants treated with increasing concentrations of AtPep1 (0.1, 1, 10, and 20 nM) (B) 24 h before infection. Infection was quantified 5 days after inoculation with 1 μl droplets of 5 × 10^3^ spores/ml of P. cucumerina BMM by a disease rating in trypan blue stained leaves, measured as a percentage of the infected leaf surface. Colors mean % of diseased leaves in a scale (0 = healthy leaves; 1 = leaves with less than 25% of diseased surface; 2 = leaves with 25–50%; 3 = leaves with 50–75% of the diseased surface, 4 = leaves with more than 75% of the surface diseased). Different letters indicate statistically significant differences (ANOVA, Fisher’s Least Significant Difference (LSD) test; P < 0.05, n = 6). The experiment had 6 plants per treatment and was repeated at least three times with similar results. [file Image_4.TIF]

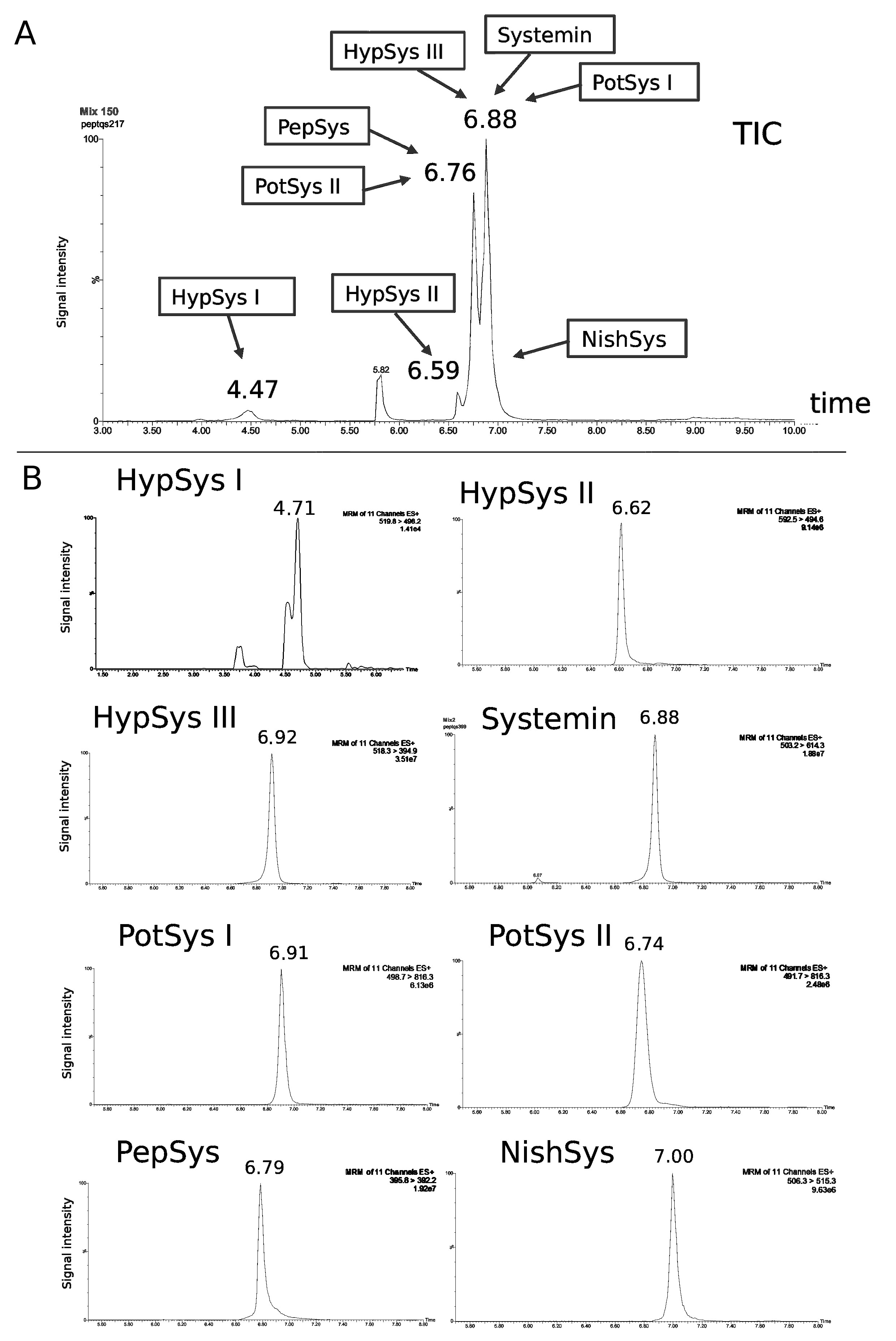

Supplement: FIGURE S5 — Peptides measured by HPLC-MS in planta. (A) total ion current (TIC) in ESI (+) of a mix of peptide standards and (B) HPLC–MS/MS chromatograms of specific transitions for each peptide of study detected in Arabidopsis plants 24 h after peptide treatment. Aliquots of 20 μl of a standard mix of 300 μl.L–1 were injected into the LC-MS system through a reversed column, at a flow rate of 0.3 ml min–1. After data recording, chromatograms were generated using the Maslynx 4.1 (Waters) software. [file Image_5.jpg]

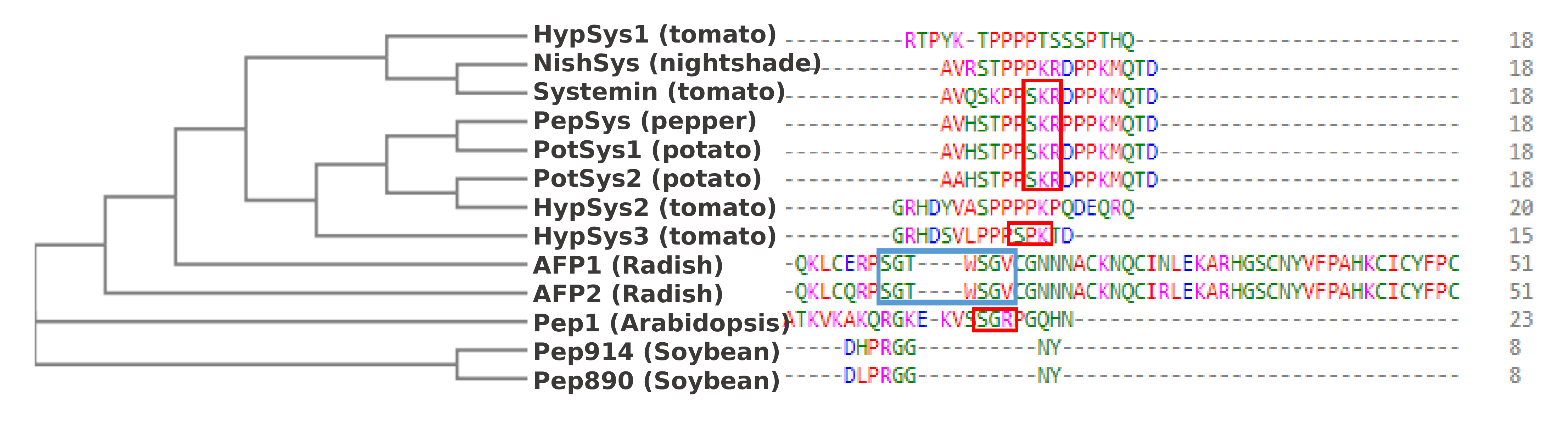

Supplement: FIGURE S6 — Peptides phylogenetic tree and multiple alignment based on their amino acid sequence. Phylogenetic tree and multiple alignment were performed using the Clustal Omega multiple alignment of the EMBL-EBI online tool (https://www.ebi.ac.uk/Tools/msa/clustalo/) using the peptides amino acid sequence provided by the Uniprot database. Numbers on the right indicate peptides’ length (number of aminoacids). Highlighted in boxes are the motifs found in each peptide using the Prosite Database (http://wwwuser.cnb.csic. es/∼pazos/cam97/). Red boxes indicate the Serine Protein Kinase C phosphorylation sites, blue box indicate N-myristoylation sites. [file Image_6.TIF]

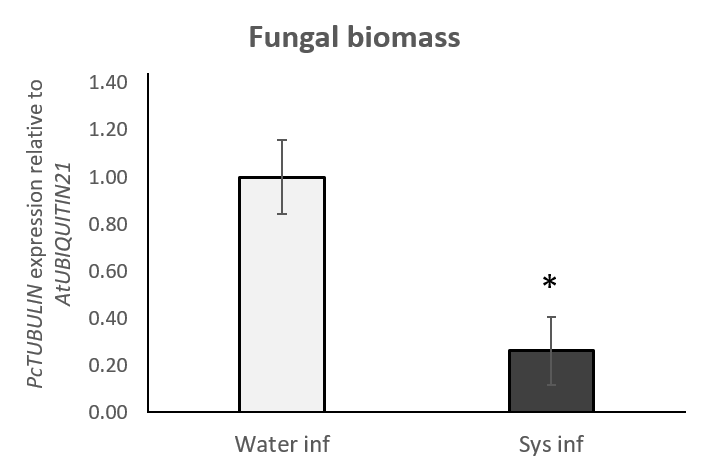

Supplement: FIGURE S7 — P. cucumerina Infection quantification by measuring fungal biomass. A ratio of PcTUBULIN relative to AtUBIQUITIN21 was calculated after performing a qPCR from gDNA of Arabidopsis infected plant samples 48 h after pathogen inoculation in watered plants and plants treated with 0.1 nM systemin 24 h before inoculation of P. cucumerina. Bars represent mean ± standard error (SD), n = 6. Asterisks mean statistical significant differences; T-test; P < 0.05, n = 6. [file Image_7.TIF]

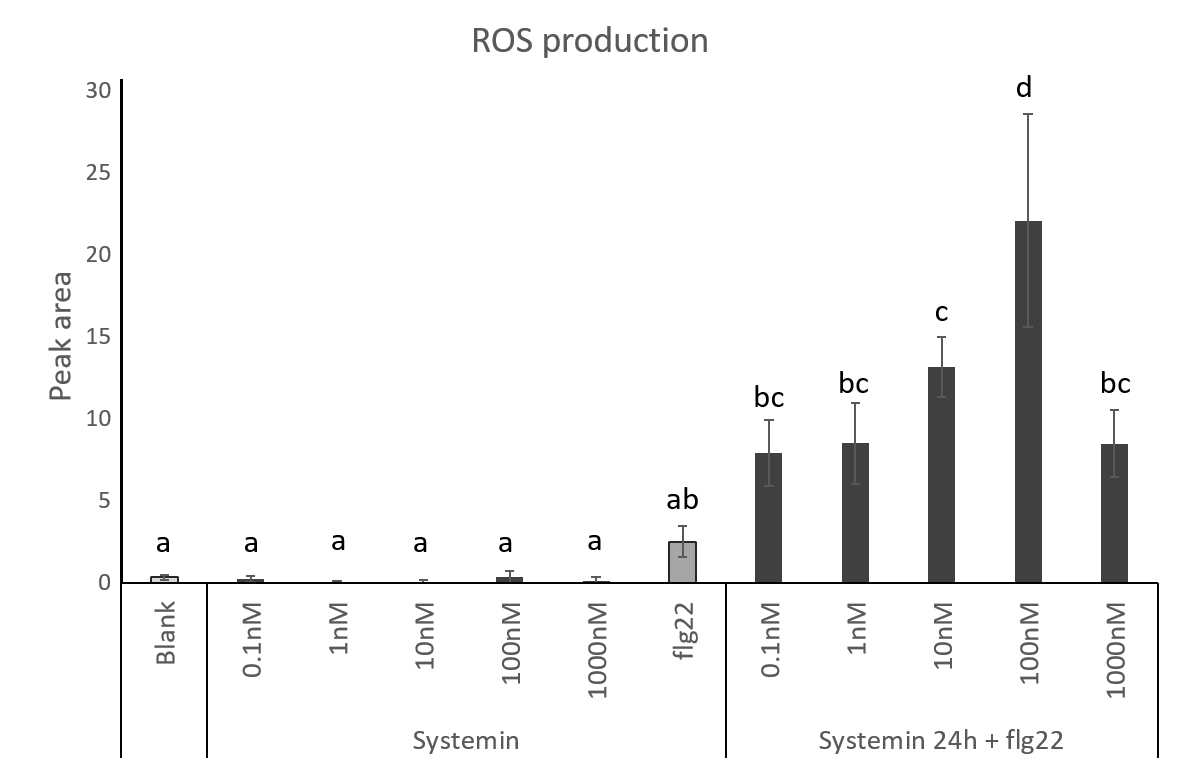

Supplement: FIGURE S8 — ROS production areas in response to Systemin and PAMP challenge. H2O2 production was measured during 1 h in leaf disks after elicitation with Systemin at different concentrations and with 100 nM flg22 in leaf disks that were pre-treated for 24 h with different concentrations of Systemin. Luminescence was expressed in Relative Luminescence Units. Bars represent means of peak areas ± standard error (SD), n = 8. Different letters represent statistically significant differences. (ANOVA, Fisher’s Least Significant Difference (LSD) test; P < 0.05, n = 8). [file Image_8.TIF]

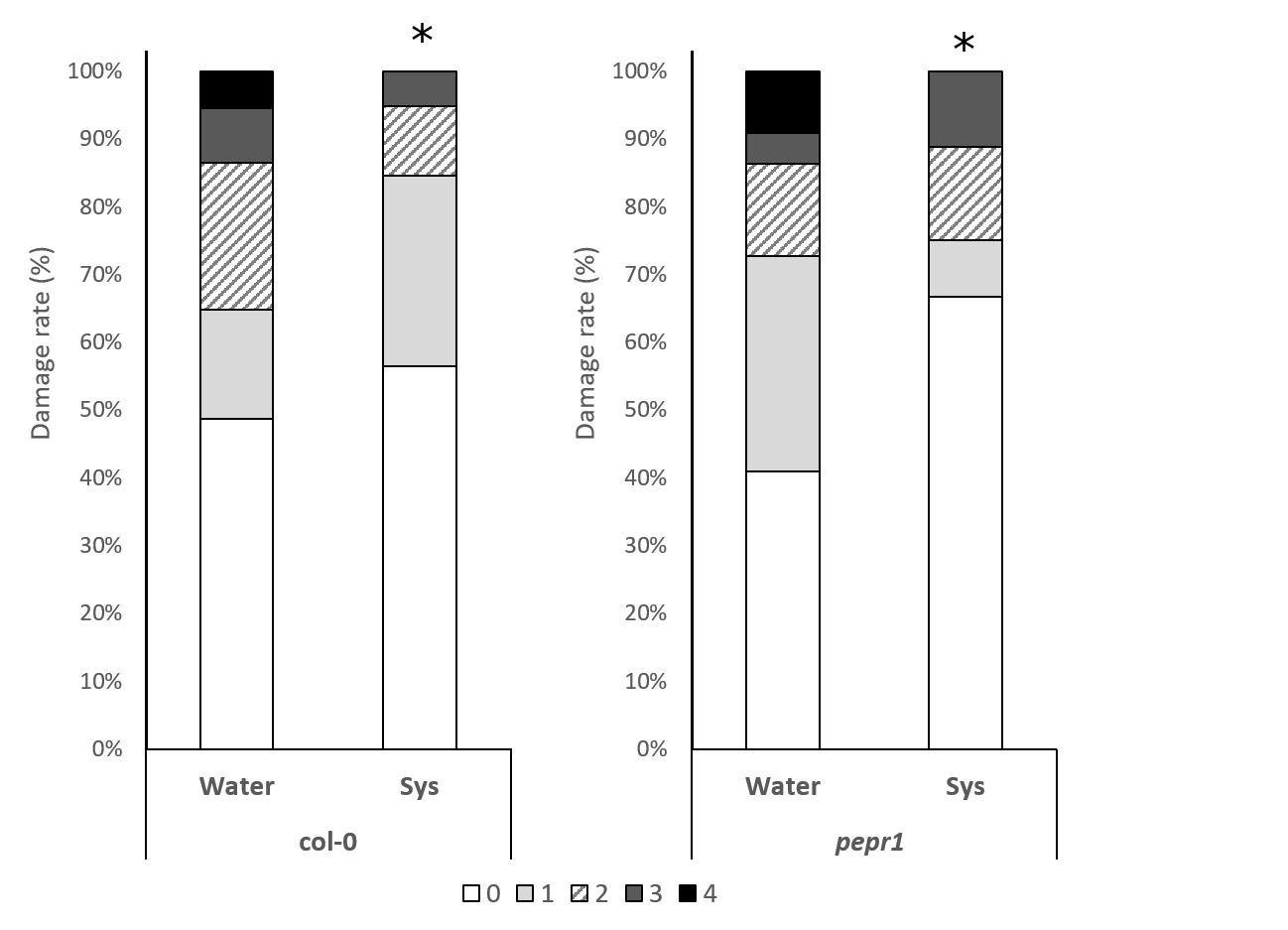

Supplement: FIGURE S9 — Sys-IR assays in the pepr1 mutant. Col-0 and pepr1 plants were challenged with 1 μl droplets of 5 × 10E3 spores/ml of P. cucumerina BMM 24 h after treatment with 0.1 nM Systemin. Infection levels were quantified 5 days after inoculation by a disease rating in trypan blue stained leaves, measured as a percentage of the infected leaf surface. Colors mean % of diseased leaves in a scale (0 = healthy leaves; 1 = leaves with less than 25% of diseased surface; 2 = leaves with 25–50%; 3 = leaves with 50–75% of the diseased surface, 4 = leaves with more than 75% of the surface diseased). Asterisks mean statistical significant differences; T-test; P < 0.05, n = 12). The experiment had 12 plants per treatment and was repeated at least three times with similar results. [file Image_9.TIF]
