# Supplementary material for: Arabidopsis Plants Sense Non-self Peptides to Promote Resistance Against Plectosphaerella cucumerina
Source: Front Plant Sci. 2020 May 8;11:529. doi: 10.3389/fpls.2020.00529 (PMC7225342; doi:10.3389/fpls.2020.00529)
Supplement: TABLE S1 — Primers used for the qPCR analysis of gene expression and Plectosphaerella cucumerina quantification. [file Table_1.DOCX]

**Supplementary Table 1.** Primers used for qPCR analysis of gene expression and *Plectosphaerella cucumerina* quantification.

| **Accession number** | **Primer name** | **Primer Sequence 5’-3’** |
| --- | --- | --- |
| AT1G74710 | ICS1 | Fw - GCGTCGTTCGGTTACAGG |
|  |  | Rv - ACAGCGAGGCTGAATCTCAT |
| AT3G45140 | LOX2 | Fw - TGATATCCGCGGCAGATCA |
|  |  | Rv - CTACCGTAACCGCTGGTCAGT |
| AT5G44420 | PDF1.2 | Fw - TTCTCTTTGCTGCTTTCGACG |
|  |  | Rv - GCATGCATTACTGTTTCCGCA |
| AT5G25760 | UBIQUITIN21 | Fw - GCTCTTATCAAAGGACCTTCGG |
|  |  | Rv - CGAACTTGAGGAGGTTGCAAAG |
| AT1G13320 | PP2A | Fw - TAACGTGGCCAAAATGATGC |
|  |  | Rv - GTTCTCCACAACCGCTTGGT |
| AT4G33430 | BAK1 | Fw - TGACGGAATTGGTGAGCTTG |
|  |  | Rv- TGTTATTAAGACGCAAGAAACGGAG |
| AT2G39660 | BIK1 | Fw - CTGGTAAGCGAGCGTTGGAT |
|  |  | Rv - TGTCTAGCCGATTGTCCACG |
| MK164271 | PcTUBULIN | Fw - CAAGTATGTTCCCCGAGCCGT |
|  |  | Rv - GGTCCCTTCGGTCAGCTCTTC |
